# Supplementary material for: Real-world effects of medications for stroke prevention in atrial fibrillation: protocol for a UK population-based non-interventional cohort study with validation against randomised trial results
Source: BMJ Open. 2021 Apr 15;11(4):e042947. doi: 10.1136/bmjopen-2020-042947 (PMC8055153; doi:10.1136/bmjopen-2020-042947)
Supplement: Supplementary data [file bmjopen-2020-042947supp001.pdf]

Appendix Table: ARISTOTLE Inclusion and Exclusion Criteria Algorithms for EHR

To be trial eligible a patient must have all inclusion criteria (IE01 to IE03)=Y and no exclusion (IE05 to IE27c)=Y

Criteria #	Used?	Criteria Text (from ARISTOTLE protocol)	Implementation Rule and Notes
		Inclusion Critera (IE01 to IE04a)	
IE01	Y	Age ≥ 18 years	Calculate age at index date, day and month of birth not available therefore calculate age by assuming birthdate=01-July-birthyear:<br>age =(indexdate-birthdate)/365.25<br><br>If age ge 18 then IE01=Y.
IE02	Y	In atrial fibrillation or atrial flutter not due to a reversible cause and documented by ECG at the time of enrollment. OR If not in atrial fibrillation/flutter at the time of enrollment, must have atrial fibrillation/flutter documented on two separate occasions, not due to a reversible cause at least 2 weeks apart in the 12 months prior to enrollment. Atrial fibrillation/flutter may be documented by ECG, or as an episode lasting at least one minute on a rhythm strip, Holter recording, or intracardiac electrogram (from an implanted pacemaker or defibrillator).	If patient has medical record corresponding to atrial fibrillation or atrial flutter on or prior to index date then IE02=Y.
		One or more of the following risk factor(s) for stroke:	IE03=Y if at least one of (IE03a, IE03b, IE03c, IE03d, IE03e) is Y.
IE03a	Y	Age 75 years or older	See IE01 for derivation of age at index date.<br>If age ge 75 then IE03a=Y
IE03b	Y	Prior stroke, TIA or systemic embolus	If patient has medical record corresponding to stroke, TIA, or systemic embolus diagnosis on or prior to index date then IE03b=Y.<br><br>Codelist search terms include 'stroke', 'cerebrovascular accident', 'cerebral infarction', 'lacunar', 'transient ischaemic attack', and synonyms for these.
IE03c	Y	Either symptomatic congestive heart failure within 3 months or left ventricular dysfunction with an LV ejection fraction (LVEF) ≤ 40% by echocardiography, radionuclide study or contrast angiography	If patient has medical record corresponding to congestive heart failure or left ventricular dysfunction diagnosis on or prior to index date then IE03c=Y.<br><br>Codelist search terms include 'heart failure', 'cardiac failure', 'congestive heart failure', 'cardiomyopathy', 'left ventricular dysfunction', 'left ventricular', 'lvef', 'new york heart association classification', 'hypertensive heart', and synonyms for these.
IE03d	Y	Diabetes mellitus	If patient has medical record corresponding to diabetes diagnosis on or prior to index date then IE03d=Y.<br><br>Codelist search terms include ‘diabetes’, both type 1 and type 2 diabetes are included.

Criteria #	Used?	Criteria Text (from ARISTOTLE protocol)	Implementation Rule and Notes
IE03e	Y	Hypertension requiring pharmacological treatment	If patient has medical record corresponding to hypertension on or prior to index date AND a prescription for an antihypertensive on or prior to index date then IE03e=Y.<br><br>Hypertension codelist search terms include 'hyperten', 'high blood pressure', ‘nephrosclerosis', and synonyms for these.
IE04	N	Women of childbearing potential (WOCBP) must be using an adequate method of contraception to avoid pregnancy throughout the treatment period of the study or for 2 weeks after the last dose of study medication, whichever is longer, in such a manner that the risk of pregnancy is minimized. WOCBP must have a negative serum or urine pregnancy test (minimum sensitivity 25 IU/L or equivalent units of HCG) within 48 hours prior to the start of investigational product.	This criteria is only partially applied - women with evidence of recent pregnancy or breastfeeding will be excluded (see IE27c).
IE04b	N	All subjects must provide signed written informed consent.	N/A for observational study
		Exclusion criteria (IE05 to IE27d)	
IE05	Y	Atrial fibrillation or flutter due to reversible causes (e.g. thyrotoxicosis, pericarditis)	If patient has medical record corresponding to reversible AF causes on or prior to index date then IE05=Y.<br><br>Codelist search terms include 'thyrotoxicosis', 'pericarditis', and synonyms for these.
IE06	Y	Clinically significant (moderate or severe) mitral stenosis	If patient has medical record corresponding to mitral stenosis on or prior to index date then IE06=Y.<br><br>Cannot determine clinical significance of ‘mitral stenosis’ terms in CPRD therefore assume if there is a record of mitral stenosis the condition is clinically significant.
IE07	Y	Increased bleeding risk that is believed to be a contraindication to oral anticoagulation (e.g. previous intracranial hemorrhage)	If patient has medical record corresponding to increased bleeding risk on or prior to index date then IE07=Y.<br><br>Codelist search terms include 'haemorrhag', 'bleed', 'aneurysm', (('intracranial' or 'brain') and ('neoplasm' or 'tumour' or 'cancer')), 'arteriovenous malformation', 'immune thrombocytopenic purpura', 'evans disease', 'hemolytic anemia', 'haemophilia', 'von willebrand disease', ('glanzmann' and 'thrombasthenia'), 'wiskott–aldrich syndrome', 'thrombocytopenia' and synonyms for these.<br><br>For some forms of more common past bleeding event such as bleeding related to menstrual or uterine bleeding, bleeding associated with surgery or injury, bleeding associated with ulcer or gastritis, eye bleeding (retinal, conjunctival) we apply the additional criteria that these must be within the last two years to be included as evidence of increased bleeding risk.

Criteria #	Used?	Criteria Text (from ARISTOTLE protocol)	Implementation Rule and Notes
IE08	Y	Conditions other than atrial fibrillation that require chronic anticoagulation (e.g. prosthetic mechanical heart valve)	If patient has medical record corresponding to a condition other than atrial fibrillation that requires chronic anticoagulation on or prior to index date then IE08=Y.<br><br>Codelist search terms include (('heart' or 'valve') and ('prosth' or 'mechanical')), 'venous thromb', and synonyms for these.
IE09	Y	Persistent, uncontrolled hypertension (systolic BP > 180 mm Hg, or diastolic BP > 100 mm Hg)	If patient has at least 2 blood pressure readings over the limit (systolic BP > 180 mm Hg, or diastolic BP > 100 mm Hg) in the 6 months prior to the index date<br>OR<br>the patient has a medical record (within 180 days prior to index date) indicating uncontrolled hypertension then IE09=Y<br><br>Codelist search terms include 'poor hypertension control', 'hypertensive crisis', 'malignant hypertension', 'severe hypertension', 'hypertension resistant to drug therapy', and synonyms for these.
IE10	Y	Active infective endocarditis	If patient has medical record corresponding to endocarditis on or prior to index date then IE10=Y.
IE11	N	Planned major surgery	N/A – do not look at future events when determining eligibility
IE12	N	Planned atrial fibrillation or flutter ablation procedure	N/A – do not look at future events when determining eligibility
IE13	N	Use of an unapproved, investigational drug or device within the past 30 days	N/A – not appropriate to apply when looking at observational data
IE14	Y	Required treatment with aspirin > 165 mg/day	If patient has a prescription for aspirin with dose > 165 mg/day and prescription data suggests drug exposure ongoing at index date then IE14=Y.<br>Note this will not pick up patients taking regular aspirin over the counter (study limitation).
IE15	Y	Simultaneous treatment with both aspirin and a thienopyridine (e.g., clopidogrel, ticlopidine)	If both aspirin and thienopyridine ongoing at index date (ie derived exposure covers index date) then IE15=Y.
IE16	Y	Severe comorbid condition with life expectancy of ≤ 1 year	If patient has medical record corresponding to a condition with a low median survival time then IE16=Y.<br>Codelist search terms include pancreatic, oesophageal, stomach, liver, gallbladder, biliary duct, bladder, lung or brain cancer, multiple myeloma, mesothelioma, CJD, and synonyms for these.
IE17	Y	Active alcohol or drug abuse, or psychosocial reasons that make study participation impractical	If patient has medical record corresponding to drug or alcohol abuse or any complications of abuse, conditions involving an impaired mental state (dementia including subtypes such as Alzheimer's), severe mental health conditions (schizophrenia, psychosis, bipolar) then IE17=Y.
IE18	Y	Recent ischemic stroke (within 7 days)	If patient has medical record corresponding to ischemic stroke within 7 days of index date (prior) then IE18=Y.

**Criteria #**	**Used?**	**Criteria Text (from ARISTOTLE protocol)**	**Implementation Rule and Notes**
IE19	Y	Severe renal insufficiency (serum creatinine > 2.5 mg/dL or a calculated creatinine clearance < 25 mL/min, See Section 6.3.2.2)	If patient has lab result showing serum creatinine > 2.5 mg/dL or a calculated creatinine clearance < 25 mL/min within 90 days prior to index date<br>OR<br>a medical record corresponding to severe renal insufficiency (chronic kidney disease stage 4 or 5, dialysis)<br>then IE19=Y
IE20	Y	ALT or AST > 2X ULN or a Total Bilirubin ≥ 1.5X ULN (unless an alternative causative factor [e.g., Gilbert's syndrome] is identified)	If patient has lab result showing ALT or AST > 2X ULN or a Total Bilirubin ≥ 1.5X ULN within 90 days prior to index date (AND no diagnosis of Gilbert's syndrome) then IE20=Y
IE21	Y	Platelet count ≤ 100,000/ mm3	If patient has lab result showing platelet count ≤ 100,000/ mm3 within 90 days prior to index date<br>OR<br>a medical record of thrombocytopenia within 90 days prior to index date<br>then IE21=Y
IE22	Y	Hemoglobin < 9 g/dL	If patient has lab result showing hemoglobin < 9 g/dL within 90 days prior to index date then IE22=Y
IE23	N	Inability to comply with INR monitoring	Patients unlikely to be able to comply with INR monitoring – evidence of drug or alcohol abuse, impaired mental state, severe mental health conditions. All these conditions are already excluded by IE17
IE24	N	Prior randomization into an apixaban clinical study	N/A
IE25	N	Prisoners or subjects who are involuntarily incarcerated	N/A
IE26	N	Subjects who are compulsorily detained for treatment of either a psychiatric or physical (e.g., infectious disease) illness	N/A
	N	Women of child bearing potential (WOCBP) unwilling or unable to use an acceptable method to avoid pregnancy:	N/A – see IE27c
IE27a	N	WOCBP using a prohibited contraceptive method	N/A

Criteria #	Used?	Criteria Text (from ARISTOTLE protocol)	Implementation Rule and Notes
IE27b	N	WOCBP include any female who has experienced menarche and who has not undergone successful surgical sterilization (hysterectomy, bilateral tubal ligation, or bilateral oophorectomy) or is not postmenopausal [defined as amenorrhea ≥ 12 consecutive months, or women on hormone replacement therapy (HRT) with documented serum follicle stimulating hormone (FSH) level > 35 mIU/mL]. Even women who are using oral contraceptives, other hormonal contraceptives (vaginal products, skin patches, or implanted or injectable products), or mechanical products such as an intrauterine device or barrier methods (diaphragm, condoms, spermicides) to prevent pregnancy, or are practicing abstinence or where their partner is sterile (e.g., vasectomy) should be considered to be of child bearing potential	N/A
IE27c	Y	Women who are pregnant or breastfeeding	Exclude women who have any medical codes relating to pregnancy (regardless of the outcome of the pregnancy), childbirth, antenatal or postnatal care, or breastfeeding in the 3 years prior to the patient's index date.
IE27d	N	Women with a positive pregnancy test on enrollment or prior to administration of investigational product.	N/A – covered by IE27c

Note: Algorithms are under development as part of this study and may be further refined prior to being finalised.
N/A = Not Applicable. For IE19-IE22 involving lab results a pragmatic approach will be taken in which a patient is assumed not to have the exclusion criteria if there is no lab result available in the 90 days prior to index date and the latest available lab result prior to index date does not meet the criteria.
