# Supplementary material for: Real-world effects of medications for stroke prevention in atrial fibrillation: protocol for a UK population-based non-interventional cohort study with validation against randomised trial results
Source: BMJ Open. 2021 Apr 15;11(4):e042947. doi: 10.1136/bmjopen-2020-042947 (PMC8055153; doi:10.1136/bmjopen-2020-042947)
Supplement: Author's manuscript [file bmjopen-2020-042947.draft_revisions.pdf]

BMJ Open

BMJ Open is committed to open peer review. As part of this commitment we make the peer review history of every article we publish publicly available.

When an article is published we post the peer reviewers' comments and the authors' responses online. We also post the versions of the paper that were used during peer review. These are the versions that the peer review comments apply to.

The versions of the paper that follow are the versions that were submitted during the peer review process. They are not the versions of record or the final published versions. They should not be cited or distributed as the published version of this manuscript.

BMJ Open is an open access journal and the full, final, typeset and author-corrected version of record of the manuscript is available on our site with no access controls, subscription charges or pay-per-view fees (http://bmjopen.bmj.com).

If you have any questions on BMJ Open's open peer review process please email info.bmjopen@bmj.com

BMJ Open

Real world effects of medications for stroke prevention in atrial fibrillation: protocol for a UK population-based non-interventional cohort study with validation against randomised trial results

Journal:	*BMJ Open*
Manuscript ID	bmjopen-2020-042947
Article Type:	Protocol
Date Submitted by the Author:	20-Jul-2020
Complete List of Authors:	Powell, Emma; London School of Hygiene and Tropical Medicine Faculty of Epidemiology and Population Health,<br>Douglas, Ian; London School of Hygiene and Tropical Medicine, Epidemiology and Population Health<br>Gungabissoon, Usha; GSK, Worldwide Epidemiology<br>Smeeth, Liam; London School of Hygiene and Tropical Medicine, Epidemiology and Population Health<br>Wing, Kevin; London School of Hygiene and Tropical Medicine,
Keywords:	EPIDEMIOLOGY, Anticoagulation < HAEMATOLOGY, Stroke < NEUROLOGY, PRIMARY CARE, STATISTICS & RESEARCH METHODS

SCHOLARONE™
Manuscripts

I, the Submitting Author has the right to grant and does grant on behalf of all authors of the Work (as defined in the below author licence), an exclusive licence and/or a non-exclusive licence for contributions from authors who are: i) UK Crown employees; ii) where BMJ has agreed a CC-BY licence shall apply, and/or iii) in accordance with the terms applicable for US Federal Government officers or employees acting as part of their official duties; on a worldwide, perpetual, irrevocable, royalty-free basis to BMJ Publishing Group Ltd ("BMJ") its licensees and where the relevant Journal is co-owned by BMJ to the co-owners of the Journal, to publish the Work in this journal and any other BMJ products and to exploit all rights, as set out in our licence.

The Submitting Author accepts and understands that any supply made under these terms is made by BMJ to the Submitting Author unless you are acting as an employee on behalf of your employer or a postgraduate student of an affiliated institution which is paying any applicable article publishing charge ("APC") for Open Access articles. Where the Submitting Author wishes to make the Work available on an Open Access basis (and intends to pay the relevant APC), the terms of reuse of such Open Access shall be governed by a Creative Commons licence – details of these licences and which Creative Commons licence will apply to this Work are set out in our licence referred to above.

Other than as permitted in any relevant BMJ Author's Self Archiving Policies, I confirm this Work has not been accepted for publication elsewhere, is not being considered for publication elsewhere and does not duplicate material already published. I confirm all authors consent to publication of this Work and authorise the granting of this licence.

For peer review only

TITLE PAGE

Title: Real world effects of medications for stroke prevention in atrial fibrillation: protocol for a UK population-based non-interventional cohort study with validation against randomised trial results

Authors:

Emma Powell[1*]

Ian Douglas[1]

Usha Gungabissoon[2]

Liam Smeeth[1]

Kevin Wing[1]

[1]Department of Non-communicable Disease Epidemiology, Faculty of Epidemiology and Population Health, London School of Hygiene and Tropical Medicine, London, UK

[2]GlaxoSmithKline, London, United Kingdom

***Submitting and corresponding author:** Emma Powell (maud.teoh@lshtm.ac.uk), London School of Hygiene and Tropical Medicine, Keppel Street, London WC1E 7HT

For peer review only

Funding statement

This work was supported by the Medical Research Council through a MRC LID studentship [grant number MR/N013638/1]

Competing interests statement

Ms Powell and Dr Wing declare no competing interests.

Professor Douglas reports grants from GlaxoSmithKline, NIHR, ABPI, MRC and holds stock in GlaxoSmithKline.

Professor Smeeth reports grants from Wellcome, MRC, NIHR, BHF, Diabetes UK, ESRC and the EU; grants and personal fees for advisory work from GSK, and historical personal fees for advisory work from AstraZeneca. He is a Trustee of the British Heart Foundation.

Ms Gungabissoon works for GlaxoSmithKline

Word count (not including abstract, tables, figures, or footnotes): 4909

Statement about prior postings and presentations: This work has not been published elsewhere.

For peer review only

Abstract

Introduction

Patients with atrial fibrillation (AF) experience an irregular heart rate and have an increased risk of stroke; prophylactic treatment with anticoagulation medication reduces this risk. Directly acting oral anticoagulants (DOACs) have been approved providing an alternative to vitamin k antagonists such as warfarin. There is interest from regulatory bodies on the effectiveness of medications in routine clinical practice; however, uncertainty remains regarding the suitability of non-interventional data for answering questions on drug effectiveness and on the most suitable methods to be used. In this study we will use data from ARISTOTLE - the pivotal trial for the DOAC apixaban - to validate non-interventional methods for assessing treatment effectiveness of anticoagulants. These methods could then be applied to analyse treatment effectiveness in people excluded from or underrepresented in ARISTOTLE.

Methods and analysis

Patient characteristics from ARISTOTLE will be used to select a cohort of patients with similar baseline characteristics from two UK electronic healthcare record (EHR) databases, CPRD Gold and Aurum (between 1 January 2013 and 31 July 2019). Methods such as propensity score matching and coarsened exact matching will be explored in matching between EHR treatment groups to determine the optimal method of obtaining a balanced cohort.[1]

Relative risk of outcomes in the EHR trial-analgous cohort will be calculated and compared with the ARISTOTLE results; if results are compatible the methods used for matching EHR treatment groups can then be used to examine drug effectiveness over a longer duration of exposure and in special patient groups of interest not studied in the trial.

Ethics and dissemination

The study has been approved by the Independent Scientific Advisory Committee of the UK Medicines and Healthcare Products Regulatory Agency. Results will be disseminated in scientific publications and at relevant conferences.

For peer review only

Article Summary

Strengths and limitations of this study

Strengths

- Selection of EHR patients matched to the randomised controlled trial (RCT) patients allows assessment of the ability of non-interventional methods to detect effectiveness of treatments for stroke prevention in AF within a RCT-analagous population.
- Combined Clinical Practice Research Database (CPRD) Gold and Aurum population broadly representative of the patients prescribed apixaban and warfarin for AF in routine clinical practice in the UK.

Limitations

- Some of the criteria that were assessed for ARISTOTLE eligibility may not be well recorded in CPRD.
- Adherence to medication will need to be assessed based on proxy variables (time covered by prescription for apixaban, INR for warfarin).
- Ascertainment of outcomes via CPRD is based on recording for clinical record keeping rather than for specifically detecting study outcomes.

For peer review only

Introduction

Background and rationale

Atrial fibrillation (AF) is a common cause of cardiac arrhythmia with symptoms including palpitations, fainting, and shortness of breath, however some patients may be asymptomatic . The prevalence of AF in the UK is estimated to be around 3%,[2] increasing from 0.2% in people aged 45-54 years to 8.0% in those 75 and older.[3] The lack of organised atrial contraction in AF can lead to the formation of thrombi, meaning that patients with AF have a five fold higher risk of stroke which is an important cause of mortality and disability.[4–6]

Current UK guidelines recommend use of prophylactic treatment with anticoagulation medication to reduce the risk of stroke. Warfarin, a vitamin K antagonist and the previous standard anticoagulant treatment, has many treatment and dietary interactions requiring frequent monitoring of a patient's International Normalised Ratio (INR), to maintain anticoagulant activity within a narrow range (2.0-3.0). Low levels put the patient at higher risk of stroke while high levels lead to a higher risk of bleeding.[7] In 2011, the first direct acting oral anticoagulant (DOAC) dabigatran was approved for the treatment of AF in the EU, it was anticipated to provide easier to manage long term anticoagulation therapy for AF patients given the complex safety profile of warfarin. ARISTOTLE, a pivotal randomised controlled trial (RCT) of the DOAC apixaban, demonstrated superiority over warfarin for both prevention of stroke and safety (major bleeding) amongst individuals with AF.[8]

The generalisability of the ARISTOTLE trial is limited by the strict eligibility criteria; evidence on apixaban's treatment effect is therefore lacking for patients who would not have met the eligibility criteria such as those with a mechanical heart valve, at increased bleeding risk, or with severe comorbid conditions. The regulatory environment now demands evidence of treatment effectiveness outside the confines of randomised trials.[9,10] Non-interventional data sources have the potential to overcome many of the RCT limitations given that they contain data for a wide spectrum of patients treated with the drug in routine care including patients who would have been excluded from trials. Data collected as a standard part of patient care such as electronic healthcare record (EHRs) provide a valuable opportunity to obtain evidence on the effectiveness of apixaban in a routine care setting. A key problem with using these data is that the absence of randomisation leaves them highly susceptible to confounding making it difficult to have confidence in the results.

To address this lack of confidence this study will apply innovative matching approaches to create a trial-analogous non-interventional cohort for analysis. Records from UK EHRs will be matched to ARISTOTLE patients before using methods for matching between treatment groups within the non-interventional EHR data, creating an EHR population similar to the trial population that is well balanced by treatment group. If successful, estimates of effectiveness and safety of apixaban

For peer review only

obtained from analysis of this ARISTOTLE-analagous cohort should be comparable with the ARISTOTLE results. The non-interventional analyses methods used to obtain these results may then be used to reliably estimate effects in under studied AF patient groups.

Aims and objectives

The aims of this study are (1) to measure the association between anticoagulation treatments for stroke prevention in AF and time to stroke, systemic embolism (SE), myocardial infarction (MI), major bleeding, and mortality amongst an ARISTOTLE-analogous cohort of patients from UK electronic health records (EHR), and (2) to develop a methodological framework with in-built validation, for using observational electronic health records to answer questions about DOAC risks and benefits in patients excluded from or underrepresented in the RCTs.

The specific objectives are:

Objective 1. Check comparability of EHR data and robustness of methods for measuring stroke prevention medication effectiveness in an ARISTOTLE-analagous cohort from EHR data by comparing with ARISTOTLE results.

Objective 2. Extension of trial findings: Measure treatment effects of apixaban in patients excluded from ARISTOTLE.

Objective 3. Comparative effectiveness: Compare treatment effectiveness between multiple individual anticoagulants (warfarin, apixaban, rivaroxaban, dabigatran) in all anticoagulant recipients (no eligibility criteria other than diagnosis of AF).

Methods and analysis

Figure 1 provides an overview of the study, covering the objectives and data sources used, and how RCT data will be used in Objective 1 to validate methods for analysing effectiveness of treatments for stroke prevention in AF in non-interventional data. Should Objective 1 prove successful the validated methods will be applied to unanswered questions in Objective 2 and 3.

Study design

We will use a retrospective cohort study design to evaluate the effects of prescribing apixaban vs warfarin and then vs other DOACs for prevention of stroke and SE in AF on key effectiveness and safety outcomes using non-interventional primary care data.

Setting/data sources

Patient data used in this study will be obtained from several sources: primary care data on UK NHS patients from CPRD Gold and Aurum databases, additional data on hospital events and mortality on

For peer review only

UK NHS patients with linked data from the Hospital Episodes Statistics (HES) and Office of National Statistics (ONS) databases, and results from the ARISTOTLE trial.

ARISTOTLE

ARISTOTLE was a randomized, double-blind trial completed in 2011, comparing apixaban with warfarin in the prevention of stroke and SE. The trial included 18,201 patients with atrial fibrillation and at least one additional risk factor for stroke. The trial was designed to test for noninferiority of apixaban compared with warfarin, and showed apixaban superiority for (1) the primary outcome of stroke or SE: hazard ratio [HR], 0.79; 95% confidence interval [CI], 0.66-0.95),[8] (2) the safety endpoint of major bleeding (HR, 0.69; 95% CI, 0.60-0.80), and (3) death from any cause (HR, 0.89; 95% CI, 0.80-0.99). The ARISTOTLE findings led to NICE guidelines on stroke prophylaxis in AF patients recommending apixaban as a treatment. Baseline patient characteristics from ARISTOTLE will be used in selection of participants in Objective 1.

CPRD Gold

CPRD Gold is a database containing anonymised data from over 625 primary care practices across the UK (approximately 13 million patient records) and is representative of the UK population with respect to age, gender and ethnicity.[11] Gold contains information on clinical diagnoses, prescribing, referrals, tests and demographic/lifestyle factors. General practices must meet prespecified standards for research-quality data to contribute data.

CPRD Aurum

CPRD Aurum contains primary care records similar to Gold but based on practices using EMIS software, whereas Gold has data from practices using Vision software. CPRD Aurum contains data on 19 million patients from 738 practices (10% of English practices) with 7 million active patients.[12]

For peer review only

Selection of participants

Participants will be selected from CPRD Gold and Aurum between 1 January 2013 and 31 July 2019. All patients will need to have been registered with a practice contributing research quality data for at least 6 months. Participant selection criteria will then vary by objective as detailed below.

Objective 1: Validation of non-interventional methods by comparing with trial results

An overview of each of the steps for participant selection for Objective 1 is provided in Figure 2.

Step 1

We will select all (HES and ONS linked) patients in the EHR cohort (CPRD Gold and Aurum) who would have met the following **inclusion** criteria for the ARISTOTLE study, at least 6 months after patient registration in the database on or prior to the index date:

- diagnosis of atrial fibrillation
- age 18+ years
- one or more stroke risk factor (age 75 years or older; prior stroke, TIA, or SE; congestive heart failure; diabetes mellitus; hypertension)

In ARISTOLE, patients randomised to apixaban were new users of apixaban whilst both treatment arms were allowed to be previous users of warfarin, with patients stratified by prior warfarin/VKA exposure. To mirror ARISTOTLE we will assess trial criteria for apixaban patients on the date of their first prescription of apixaban whilst allowing patients prescribed warfarin to become eligible at any warfarin prescription date during the study period. We will then exclude patients who meet any of the following ARISTOTLE study **exclusion** criteria prior to their eligible-for-inclusion date:

- AF due to reversible causes
- mitral stenosis
- increased bleeding risk
- conditions other than AF requiring chronic anticoagulation
- persistent, uncontrolled hypertension
- active infective endocarditis
- current treatment with aspirin > 165 mg/day
- simultaneous current treatment with both aspirin and a thienopyridine
- conditions likely to interfere with participation in the trial or cause death within 1 year
- recent alcohol or drug abuse, or psychosocial reasons making study participation impractical
- recent ischemic stroke (within 7 days)
- severe renal insufficiency
- ALT or AST > 2X ULN or Total Bilirubin ≥ 1.5X ULN
- platelet count ≤ 100,000/ mm3
- haemoglobin < 9 g/dL
- pregnancy or breastfeeding

Feasibility counts in Gold found approximately 60% of AF patients prescribed apixaban met the ARISTOTLE trial criteria.

For peer review only

Step 2

We will select a subset of apixaban patients from our EHR pool to create a cohort that matches the ARISTOTLE apixaban participants on a selection of the following baseline characteristics:

- Age
- Sex
- BMI
- Systolic blood pressure
- Congestive heart failure or left ventricular systolic dysfunction
- Hypertension requiring treatment
- Diabetes mellitus
- Prior stroke/thromboembolism
- Smoking status
- Alcohol consumption
- Level of renal impairment
- Prior VKA/warfarin use
- Concomitant use of: aspirin, antiplatelet or NSAID, lipid lowering drug therapy, or CYP3A4 inhibitor

This step will generate a group of ARISTOTLE-analogous apixaban patients, with similar baseline characteristics to ARISTOTLE subjects at the point of randomisation (n~9,000).

The variables selected are expected to influence the likelihood of the outcomes of interest. Exact selection of matching variables will depend on the quality and completeness of the data available and a balance will be struck between matched sample size and balance. Different methods to facilitate selection of a matched cohort will be explored, such as propensity score matching (PSM) and coarsened exact matching (CEM),[1] a nonparametric method that may give estimates with lower variance and bias for a given sample size compared than other methods.[13]

Step 3

The resulting trial matched sample of EHR apixaban patients will be matched to the warfarin ARISTOTLE-eligible EHR patients (Figure 2) using a matching method such as PSM, or CEM (final method selected based upon giving optimal sample size versus balance). The covariates for consideration in matching between EHR treatment arms or construction of a PS model will include the variables listed above in step 2 along with additional EHR variables such as data source (Gold or Aurum), socioeconomic status, and comorbidities. Each apixaban patient from the ARISTOTLE-eligible EHR patients will be matched 1:1 with the warfarin EHR patient with the closest match giving a trial-analogous cohort of ~18,000.

Step 4

The hazard ratio for the outcomes of interest (time to: stroke/SE, MI, major bleeding, and mortality) will then be calculated. For the primary outcome (time to stroke/SE) the EHR results will be validated against the ARISTOTLE trial results using the criteria detailed in Statistical Analysis (Validation of Observational Results Against Aristotle Data).

For peer review only

Objective 2: we will select patients who would not have been included in ARISTOTLE (and therefore would not have been included in the Objective 1 cohort) or who are under-represented in ARISTOTLE. Specifically, this will include patient groups such as patients with an AF diagnosis in the EHR cohort meeting these additional criteria:

- no evidence of at least one additional risk factor for stroke

OR

- AF due to reversible causes

OR

- evidence of drug/alcohol abuse

OR

- severe comorbid condition: disease with a likelihood of causing death within 1 year or reasons making participation unpractical (such as dementia)

In these special patient populations the same outcomes as objective 1 will be assessed.

Objective 3: we will select all patients with AF who have a prescription for apixaban, warfarin, rivaroxaban, or dabigatran in the treatment period. The same outcomes as objective 1 will be assessed with patients stratified on whether they would have met ARISTOTLE trial criteria.

Exposures, outcomes and co-variates

Exposures

For all objectives, exposures will be determined using CPRD Gold and Aurum prescribing records and code lists for anticoagulant treatments with no restrictions placed on the dose prescribed.

For Objectives 1 and 2, use of apixaban is the primary exposure of interest and will be compared with warfarin.

For Objective 3 other stroke prevention treatments for AF will also be compared, namely dabigatran and rivaroxaban.

Outcomes

Outcomes to be measured are as follows:

1. Stroke (ischemic or hemorraghic) or systemic embolism
2. Major bleeding
3. Myocardial Infarction
4. All cause mortality
5. Time to AF treatment change

Outcomes will be ascertained using a combination of CPRD, HES, and ONS data.

Covariates

For peer review only

The variables to be considered for matching patients are detailed in the selection of participants for Objective 1 (Step 2).

Sample size

Objective 1

ARISTOTLE included 9,120 patients in the apixaban arm therefore it was estimated a minimum of 15,000 EHR apixaban patients were needed for matching to be feasible. In CPRD Gold approximately 8,400 patients were eligible (January 2018). Aurum (June 2019) contained 23,526 AF apixaban patients not registered in practices that had previously contributed data to Gold. Assuming the proportion of Aurum patients meeting ARISTOTLE eligibility criteria would be similar to the proportion in Gold (~60%) gave an estimate of 14,115 trial eligible apixaban patients. Combining Gold and Aurum is therefore estimated to give >22,000 unique trial-eligible EHR apixaban patients.

Objectives 2 and 3

From feasibility counts we are confident we will have sufficient numbers of patients to allow well-powered analyses for objectives 2 and 3. For example, we estimate the number of people with no evidence of at least one additional risk factor for stroke for objective 2 would be >3000 people in each exposure group.

Statistical analysis

Methods of Analysis

ARISTOTLE used an intent-to-treat (ITT) approach for the primary efficacy analysis, and an on-treatment approach for sensitivity analysis and safety outcomes. We will perform equivalent analyses by using 2 different censoring schemes: a primary censoring scheme censoring 5 years after index date (reflecting the maximum possible follow-up in ARISTOTLE) for the primary effectiveness analyses, and an on-treatment scheme censoring around time of last study drug for the sensitivity analysis and safety outcome. For the on-treatment censoring scheme date of last exposure will be estimated using patient prescription data - to allow for drug half-life, stockpiling of tablets and less than 100% adherence we will add 30 days after the apparent end of treatment.

Demographic and baseline variables will be presented before and after matching steps. As the primary analysis accounts neither for treatment switching nor discontinuation, the proportion of patients discontinuing treatment and time to treatment discontinuation will be tabulated.

The primary effectiveness endpoint is time to first occurrence of confirmed stroke (ischemic, hemorrhagic, or unspecified type) or SE during the study, regardless of whether the subject is receiving treatment at the time (primary censoring scheme). Comparisons will be made according to prescribed treatment (apixaban vs warfarin).

For peer review only

All time to event endpoints will be analysed using a Cox proportional hazards model including treatment group as a covariate and prior warfarin/VKA status (experienced, naïve). Point estimates and two-sided 95% CIs will be constructed for the outcome.

Secondary outcomes cover the key safety outcome of major bleeding and the individual outcomes of stroke, SE, MI, and mortality. Secondary outcomes other than major bleeding will use the ITT censoring scheme, major bleeding will use the on-treatment censoring scheme.

Validation of Results Against Aristotle Data

In Objective 1 alone we will validate the findings from our primary analysis against ARISTOTLE by determining whether results are compatible with the trial results. ARISTOTLE demonstrated superiority of apixaban over warfarin for the primary endpoint (HR 0.79, 95% CI 0.66-0.95).[8] The treatment effect seen with EHR data may be weaker than that seen in ARISTOTLE.

An analysis of EU patients in ARISTOTLE showed a smaller treatment difference for the primary endpoint and death: HR (95% CI) for stroke/SE 0.92 (0.56-1.52), all cause death 0.89 (0.68-1.18). The European Medicines Agency Assessment Report suggested the smaller treatment effect may have been due to superior INR control in the warfarin arm of the EU subgroup (median TTR 68.93%);[14] this study could provide additional evidence on this point.

Either a result of superiority or non-inferiority will be considered compatible with ARISTOTLE results. We have set two criteria that must be met to conclude results are consistent with the trial result:

1. The effect size must be clinically comparable with the ARISTOTLE findings; the hazard ratio for time to stroke/SE with the EHR must be between 0.69 and 0.99. This range is not symmetrical around the ARISTOTLE estimate of 0.79 as it is anticipated the treatment effect in routine clinical care may be weaker than that seen in the optimised setting of a clinical trial.
2. The upper limit of the 95% CI for the rate ratio must be less than 1.52 (upper limit in the EU subgroup of ARISTOTLE).

In addition, if the upper limit of the 95% CI is less than 1 then superiority of apixaban vs warfarin will be concluded.

Sensitivity analyses

Primary and secondary effectiveness outcomes will also be analysed using the on-treatment censoring scheme to investigate whether the extent of treatment discontinuation compromises confidence in the effectiveness analyses.

Exclusion of patient-time post treatment discontinuation in the safety and sensitivity analyses might bias results towards a conclusion of no difference;[15] the set of patients who switch or discontinue treatment will therefore be examined to ascertain whether biases of this nature may have occurred.

For peer review only

Additional analyses may be performed using methods such as inverse-probability-of-censoring weighting or a rank-preserving structural failure time model to estimate the treatment effect that would have been observed in the absence of treatment switching.

Adherence will be estimated in the EHR cohort to enable comparisons with the trial and investigate the extent to which this may have influenced differences in treatment effect observed. For apixaban we will calculate the proportion of days covered (PDC) over a patient's time when on treatment as a measure of adherence. Warfarin dose is poorly recorded in EHR therefore warfarin adherence will be estimated by looking at adherence to other long-term daily medications as a proxy measure and by looking at INR control by calculating percent Time INR in Therapeutic Range (TTR) as a measure of overall warfarin treatment regime adherence.

We will perform a supplementary analysis in patients deemed adherent (PDC ≥ 80% matching ARISTOTLE compliance limit) along with an exploratory subgroup analysis by INR TTR.

Plan for addressing confounding

In the study period apixaban was a newly available treatment leading to the possibility of channelling bias. For objective 1 by applying trial eligibility criteria to both treatment cohorts and matching using the baseline covariates we should avoid channelling bias. To handle confounding treatment arms will be matched using the optimal method selected. Unmeasured or unknown confounding may remain and this will be explored in the analysis and discussion of results.

Missing Baseline Data

UK EHR data have been shown to be almost complete for drug prescribing and information on important comorbidity are well recorded. For some variables such as renal function and alcohol intake, a patient is more likely to have no data entered if there is no overt clinical evidence of abnormality; in such cases we may take a pragmatic approach categorising into a parameter (“evidence of” vs “no evidence of”) with those with no data included in the “no evidence of” group. For BMI and SBP we cannot assume data are missing at random as we expect a patient is less likely to have these recorded if they appear healthy weight and do not have hypertension respectively or if they have a lower comorbidity burden. Patients with missing BMI or SBP will therefore be excluded from the trial-eligible cohort.

Missing Prescription Data

Treatment may be initiated in secondary care meaning the first prescription of patients newly initiating treatment or switching treatments are missing; to account for this we will perform a sensitivity analysis where those newly initiating treatment are assigned an earlier derived index date. Hospitalised patients may have prescriptions in secondary care leading to treatment gaps in their primary care data. We will investigate the occurrence of hospitalisation around treatment

For peer review only

discontinuation and assess the potential impact on the results of missed events by performing a sensitivity analysis with different extended derived dates of last dose. Some concomitant drugs used in determining eligibility and matching patients are available over the counter meaning we may miss that patients are exposed to these; we expect OTC use of these drugs to be similar in both treatment groups.

Missing Outcome Data

EHR data are shown to be almost complete for mortality.[16] Patient deaths missing from EHRs are expected to be missing at random equally in both treatment arms thereby not altering the overall direction of treatment effect. The classification of unspecified stroke type will cause uncertainty in the main safety endpoint and may lead to a lower event rate for major bleeding compared with the trial; this would affect the power but should not affect the treatment effect seen as events are expected to be missing at random from both treatment arms.

Limitations of the study design, data sources, and analytic methods

Some of the criteria assessed for ARISTOTLE eligibility may not be well recorded in CPRD, criteria such as alcohol and drug abuse may not be captured for all patients. For criteria such as “increased bleeding risk” it is unclear which codes to include and time scale to consider. These limitations are consistent with our aim to select a population as similar as possible to ARISTOTLE whilst acknowledging differences will remain. The most important risk factors for the primary outcome of stroke (the components of CHA2DS2-VASc score for AF stroke risk) are mostly well recorded in CPRD.[17]

There are differences in the coding systems used by the two EHR data sources and completeness of coding may differ between the two; the potential impact of this will be ascertained by comparisons of rates of diagnoses, baseline variables, and prescriptions of interest. Inclusion of data source as a matching variable should prevent discrepancy between the sources from biasing results. We will explore different methods of combining Gold and Aurum, namely analysing separately by database and combining the results as a metanalysis as an alternative to combining data before analysis.

The main focus of the study is validation of our methodology through assembling a cohort of patients comparable to the patients in ARISTOTLE and finding similar results to the trial. Criteria to determine the success of the methodology have been pre-specified in the protocol. Given the use of CPRD data to determine treatment effectiveness is not yet well established, a finding that these data are not suitable to answer questions on intended effectiveness will be a useful conclusion.

For peer review only

Ethics and dissemination

Approval by ethics and scientific comittees

An application for scientific approval related to use of CPRD data was approved by the Independent Scientific Advisory Committee (ISAC) of the Medicines and Healthcare Products Regulatory Agency (MHRA).

Dissemination plans

The results of the study will be submitted to peer reviewed journals and presented at conferences. Relevant charities will be contacted for guidance on dissemination of results to patients in an accessible manner. We will communicate with NICE to convey any results relevant to the guidance they have issued on AF, and with the MHRA if findings may impact the risk/benefit profile of anticoagulation treatments in AF patients.

For peer review only

References

1 Iacus SM, King G, Porro G. Causal Inference without Balance Checking: Coarsened Exact Matching. *Polit Anal* 2012;**20**:1–24. doi:10.1093/pan/mpr013

2 Adderley NJ, Ryan R, Nirantharakumar K, *et al.* Prevalence and treatment of atrial fibrillation in UK general practice from 2000 to 2016. *Heart* 2019;**105**:27–33. doi:10.1136/HEARTJNL-2018-312977

3 Davis RC, Hobbs FDR, Kenkre JE, *et al.* Prevalence of atrial fibrillation in the general population and in high-risk groups: the ECHOES study. *Europace* 2012;**14**:1553–9. doi:10.1093/europace/eus087

4 Wolf PA, Abbott RD, Kannel WB. Atrial fibrillation as an independent risk factor for stroke: The framingham study. *Stroke* 1991;**22**:983–8. doi:10.1161/01.STR.22.8.983

5 Brønnum-Hansen H, Davidsen M, Thorvaldsen P. Long-term survival and causes of death after stroke. *Stroke* 2001;**32**:2131–6. doi:10.1161/hs0901.094253

6 Adamson J, Beswick A, Ebrahim S. Is stroke the most common cause of disability? *J Stroke Cerebrovasc Dis* 2004;**13**:171–7. doi:10.1016/j.jstrokecerebrovasdis.2004.06.003

7 Nelson WW, Wang L, Baser O, *et al.* Out-of-range INR values and outcomes among new warfarin patients with non-valvular atrial fibrillation. *Int J Clin Pharm* 2014;**37**:53–9. doi:10.1007/s11096-014-0038-3

8 Granger CB, Alexander JH, McMurray JJV, *et al.* Apixaban versus Warfarin in Patients with

Atrial Fibrillation. *N Engl J Med* 2011;**365**:981–92. doi:10.1056/NEJMoa1107039

9 U.S. Department of Health and Human Services F and D. Guidance for Industry Postmarketing Studies and Clinical Trials — Implementation of Section 505(o)(3) of the Federal Food, Drug, and Cosmetic Act.

10 EUROPEAN PARLIAMENT AND THE COUNCIL OF THE EUROPEAN UNION. REGULATION (EU) No 1235/2010 OF THE EUROPEAN PARLIAMENT AND OF THE COUNCIL of 15 December 2010.

11 Herrett E, Gallagher AM, Bhaskaran K, *et al.* Data Resource Profile: Clinical Practice Research Datalink (CPRD). *Int J Epidemiol* 2015;**44**:827–36. doi:10.1093/ije/dyv098

12 Wolf A, Dedman D, Campbell J, *et al.* Data resource profile: Clinical Practice Research Datalink (CPRD) Aurum. *Int J Epidemiol* Published Online First: 11 March 2019. doi:10.1093/ije/dyz034

13 King G, Nielsen R, Coberley C, *et al.* Comparative Effectiveness of Matching Methods for Causal Inference. https://gking.harvard.edu/publications/comparative-effectiveness-matching-methods-causal-inference (accessed 4 Sep 2018).

14 European Medicines Agency. Committee for Medicinal Products for Human Use (CHMP). Assessment report Eliquis apixaban. Procedure No.:EMEA/H/C/002148/X/04/G. EMA/641505/2012. 2012.

15 Jones B, Jarvis P, Lewis JA, *et al.* Trials to assess equivalence: the importance of rigorous methods. *BMJ* 1996;**313**:36–9.http://www.ncbi.nlm.nih.gov/pubmed/8664772 (accessed 4 Sep 2018).

16 Gallagher AM, Williams T, Leufkens HGM, *et al.* The Impact of the Choice of Data Source in Record Linkage Studies Estimating Mortality in Venous Thromboembolism. *PLoS One* 2016;**11**:e0148349. doi:10.1371/journal.pone.0148349

17 Khan NF, Harrison SE, Rose PW. Validity of diagnostic coding within the General Practice Research Database: a systematic review. *Br J Gen Pract* 2010;**60**:e128–36. doi:10.3399/bjgp10X483562

18 Wing K, Williamson E, Carpenter JR, *et al.* Real-world effects of medications for chronic obstructive pulmonary disease: protocol for a UK population-based non-interventional cohort study with validation against randomised trial results. *BMJ Open* 2018;**8**:e019475. doi:10.1136/bmjopen-2017-019475

For peer review only

Authors statement

EP, KW, ID, UG, LS contributed to study question and design. EP wrote the first draft of the protocol manuscript (based upon original proposal to MRC, ISAC that EP, KW, ID, UG and LS all contributed to). EP, KW, ID, UG, LS contributed to further drafts and approved the final version.

Acknowledgements

No information for this section.

Data statement

There are currently no unpublished data from this study as it is a protocol.

All of the data sources described can be accessed by making formal applications to the owners of the data (CPRD/HES data for the routinely collected non-interventional data and Bristol-Myers Squibb for the trial summary and results used for validation of non-interventional methods).

Figure 1: Overview of study objectives and sources of data for the real-world effects of medications for stroke prevention in AF study

(RCT=randomised controlled trial, CPRD= the UK Clinical Practice Research Datalink, AF=Atrial Fibrillation, EHR= Electronic Healthcare Records)

ref: Adapted from Figure 1 “Real-world effects of medications for chronic obstructive pulmonary disease: protocol for a UK population-based non-interventional cohort study with validation against randomised trial results” (Wing et al).[18]

Figure 2: Flowchart illustrating the Assembly of a Matched Trial-analogous Cohort of EHR Patients

EHR= Electronic Health Record; CPRD= Clinical Practice Research Datalink; AF= Atiral fibrillation

For peer review only

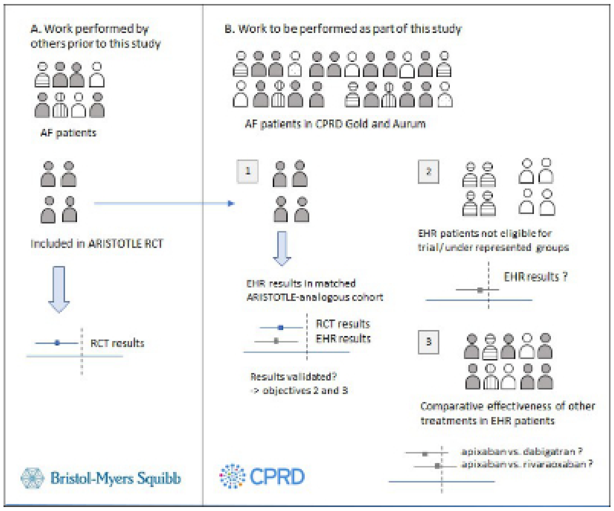

A. Work performed by others prior to this study

ARISTOTLE: RCT that investigated effectiveness and safety of apixaban vs warfarin in prevention of stroke and systemic embolism in AF patients. RCTs results inform clinical practice despite only a subset (based on trial inclusion and exclusion criteria) of the total population of AF patients being included in the RCTs of stroke prophylaxis treatments.

B. Work to be performed as part of this study

1. Objective 1

A cohort of ARISTOTLE-analogous patients will be selected from UK EHRs (CPRD Gold and Aurum), by matching EHR patients prescribed apixaban to the apixaban patients included in the trial on baseline characteristics. EHR patients prescribed warfarin will then be matched to the trial-analagous EHR apixaban patients. An analysis of the effectiveness of apixaban vs. warfarin on prevention of stroke/systemic embolism will then be performed on this ARISTOTLE-analagous EHR cohort. If the results obtained are comparable to those obtained in ARISTOTLE, this will serve as a validation step, showing that data from the non-interventional CPRD Gold and Aurum sources can reliably be used to study stroke prevention treatment effects in AF.

2. Objective 2

The validated analysis techniques used for Objective 1 will then be used to study UK EHR patients who would not have been eligible for inclusion in an RCT or are under-represented in RCTs due to their age or presence of other comorbidities, for whom the comparative effects of anticoagulants in stroke prevention in AF is unclear.

3. Objective 3

The validated analysis techniques used for Objective 1 will then be used to compare effectiveness of apixaban vs rivaroxaban and apixaban vs dabigatran.

Figure 1: Overview of study objectives and sources of data for the real-world effects of medications for stroke prevention in AF study
(RCT=randomised controlled trial, CPRD= the UK Clinical Practice Research Datalink, AF=Atrial Fibrillation, EHR= Electronic Healthcare Records)
ref: Adapted from Figure 1 “Real-world effects of medications for chronic obstructive pulmonary disease: protocol for a UK population-based non-interventional cohort study with validation against randomised trial results” (Wing et al).[18]

173x96mm (96 x 96 DPI)

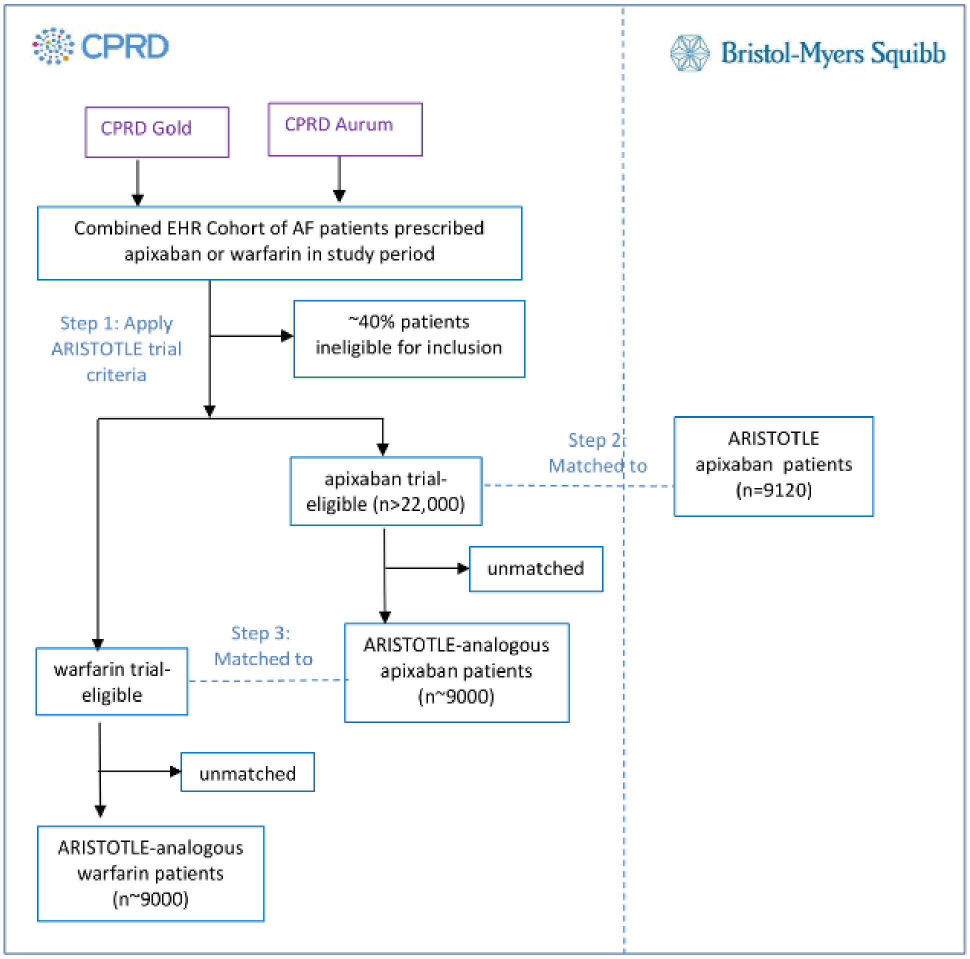

Figure 2: Flowchart illustrating the Assembly of a Matched Trial-analogous Cohort of EHR Patients
EHR= Electronic Health Record; CPRD= Clinical Practice Research Datalink; AF= Atiral fibrillation

173x171mm (96 x 96 DPI)

BMJ Open

Real world effects of medications for stroke prevention in atrial fibrillation: protocol for a UK population-based non-interventional cohort study with validation against randomised trial results

Journal:	*BMJ Open*
Manuscript ID	bmjopen-2020-042947.R1
Article Type:	Protocol
Date Submitted by the Author:	26-Nov-2020
Complete List of Authors:	Powell, Emma; London School of Hygiene and Tropical Medicine Faculty of Epidemiology and Population Health,<br>Douglas, Ian; London School of Hygiene and Tropical Medicine, Epidemiology and Population Health<br>Gungabissoon, Usha; GSK, Worldwide Epidemiology<br>Smeeth, Liam; London School of Hygiene and Tropical Medicine, Epidemiology and Population Health<br>Wing, Kevin; London School of Hygiene and Tropical Medicine,
**Primary Subject Heading**:	Epidemiology
Secondary Subject Heading:	Cardiovascular medicine, Research methods, Neurology
Keywords:	EPIDEMIOLOGY, Anticoagulation < HAEMATOLOGY, Stroke < NEUROLOGY, PRIMARY CARE, STATISTICS & RESEARCH METHODS

SCHOLARONE™
Manuscripts

I, the Submitting Author has the right to grant and does grant on behalf of all authors of the Work (as defined in the below author licence), an exclusive licence and/or a non-exclusive licence for contributions from authors who are: i) UK Crown employees; ii) where BMJ has agreed a CC-BY licence shall apply, and/or iii) in accordance with the terms applicable for US Federal Government officers or employees acting as part of their official duties; on a worldwide, perpetual, irrevocable, royalty-free basis to BMJ Publishing Group Ltd ("BMJ") its licensees and where the relevant Journal is co-owned by BMJ to the co-owners of the Journal, to publish the Work in this journal and any other BMJ products and to exploit all rights, as set out in our licence.

The Submitting Author accepts and understands that any supply made under these terms is made by BMJ to the Submitting Author unless you are acting as an employee on behalf of your employer or a postgraduate student of an affiliated institution which is paying any applicable article publishing charge ("APC") for Open Access articles. Where the Submitting Author wishes to make the Work available on an Open Access basis (and intends to pay the relevant APC), the terms of reuse of such Open Access shall be governed by a Creative Commons licence – details of these licences and which Creative Commons licence will apply to this Work are set out in our licence referred to above.

Other than as permitted in any relevant BMJ Author's Self Archiving Policies, I confirm this Work has not been accepted for publication elsewhere, is not being considered for publication elsewhere and does not duplicate material already published. I confirm all authors consent to publication of this Work and authorise the granting of this licence.

For peer review only

TITLE PAGE

Title: Real world effects of medications for stroke prevention in atrial fibrillation: protocol for a UK population-based non-interventional cohort study with validation against randomised trial results

Authors:

Emma Powell[1*]

Ian Douglas[1]

Usha Gungabissoon[2]

Liam Smeeth[1]

Kevin Wing[1]

[1]Department of Non-communicable Disease Epidemiology, Faculty of Epidemiology and Population Health, London School of Hygiene and Tropical Medicine, London, UK

[2]GlaxoSmithKline, London, United Kingdom

***Submitting and corresponding author:** Emma Powell (maud.teoh@lshtm.ac.uk), London School of Hygiene and Tropical Medicine, Keppel Street, London WC1E 7HT

For peer review only

Word count (not including abstract, tables, figures, or footnotes): 4702

Statement about prior postings and presentations: This work has not been published elsewhere.

For peer review only

Abstract

Introduction

Patients with atrial fibrillation (AF) experience an irregular heart rate and have an increased risk of stroke; prophylactic treatment with anticoagulation medication reduces this risk. Directly acting oral anticoagulants (DOACs) have been approved providing an alternative to vitamin k antagonists such as warfarin. There is interest from regulatory bodies on the effectiveness of medications in routine clinical practice; however, uncertainty remains regarding the suitability of non-interventional data for answering questions on drug effectiveness and on the most suitable methods to be used. In this study we will use data from ARISTOTLE - the pivotal trial for the DOAC apixaban - to validate non-interventional methods for assessing treatment effectiveness of anticoagulants. These methods could then be applied to analyse treatment effectiveness in people excluded from, or underrepresented in ARISTOTLE.

Methods and analysis

Patient characteristics from ARISTOTLE will be used to select a cohort of patients with similar baseline characteristics from two UK electronic healthcare record (EHR) databases, CPRD Gold and Aurum (between 1 January 2013 and 31 July 2019). Methods such as propensity score matching and coarsened exact matching will be explored in matching between EHR treatment groups to determine the optimal method of obtaining a balanced cohort.

Absolute and relative risk of outcomes in the EHR trial-analgous cohort will be calculated and compared with the ARISTOTLE results; if results are deemed compatible the methods used for matching EHR treatment groups can then be used to examine drug effectiveness over a longer duration of exposure and in special patient groups of interest not studied in the trial.

Ethics and dissemination

The study has been approved by the Independent Scientific Advisory Committee of the UK Medicines and Healthcare Products Regulatory Agency. Results will be disseminated in scientific publications and at relevant conferences.

For peer review only

Article Summary

Strengths and limitations of this study

Strengths

- Selection of EHR patients matched to the randomised controlled trial (RCT) patients allows assessment of the ability of non-interventional methods to detect effectiveness of treatments for stroke prevention in AF within a RCT-analagous population.
- Combined Clinical Practice Research Database (CPRD) Gold and Aurum population broadly representative of the patients prescribed apixaban and warfarin for AF in routine clinical practice in the UK.

Limitations

- Some of the criteria that were assessed for ARISTOTLE eligibility may not be well recorded in CPRD.
- Adherence to medication will need to be assessed based on proxy variables (time covered by prescription forthe DOACs, time in therapeutic range based on INR measurements for warfarin); the different nature of these proxy variables means the adherence estimates may not be comparable.
- Ascertainment of outcomes via CPRD is based on recording as part of routine clinical care rather than for specifically detecting study outcomes.

For peer review only

Introduction

Background and rationale

Atrial fibrillation (AF) is a common cause of cardiac arrhythmia with symptoms including palpitations, fainting, and shortness of breath, however some patients may be asymptomatic . The prevalence of AF in the UK is estimated to be around 3%,[1] increasing from 0.2% in people aged 45-54 years to 8.0% in those 75 and older.[2] The lack of organised atrial contraction in AF can lead to the formation of thrombi, meaning that patients with AF have a five-fold higher risk of stroke which is an important cause of morbidity and mortality.[3–5]

Current UK guidelines recommend use of prophylactic treatment with anticoagulation medication to reduce the risk of stroke. Warfarin, a vitamin K antagonist and the previous standard anticoagulant treatment, has many treatment and dietary interactions requiring frequent monitoring of a patient's International Normalised Ratio (INR), to maintain anticoagulant activity within a narrow range (2.0-3.0). Low levels put the patient at higher risk of stroke while high levels lead to a higher risk of bleeding.[6] In 2011, the first direct acting oral anticoagulant (DOAC) dabigatran was approved for the treatment of AF in the EU, it was anticipated to provide easier to manage long-term anticoagulation therapy for AF patients given the complex safety profile of warfarin. ARISTOTLE, a pivotal randomised controlled trial (RCT) of the DOAC apixaban, demonstrated superiority over warfarin for both prevention of stroke and safety (major bleeding) amongst individuals with AF.[7]

The generalisability of the ARISTOTLE trial is limited by the strict eligibility criteria; evidence on apixaban's treatment effect is therefore lacking for patients who would not have met the eligibility criteria such as those at increased bleeding risk or with severe comorbid conditions. The regulatory environment now demands evidence of treatment effectiveness outside the confines of randomised trials.[8,9] Non-interventional data sources have the potential to overcome many of the RCT limitations given that they contain data for a wide spectrum of patients treated with the drug in routine care, including patients who would have been not eligible for trials. Data collected as part of routine patient care such as electronic healthcare record (EHRs) provide a valuable opportunity to obtain evidence on the effectiveness of apixaban in a routine care setting. A key problem with using these data is that the absence of randomisation leaves them highly susceptible to confounding making it difficult to have confidence in the results.

To address this lack of confidence, this study will apply innovative matching approaches to create a trial-analogous non-interventional cohort for analysis. Records from UK EHRs will be matched to ARISTOTLE patients before using methods for matching between treatment groups within the non-interventional EHR data, creating an EHR population similar to the trial population that is well balanced by treatment group. If successful, estimates of effectiveness and safety of apixaban

For peer review only

obtained from analysis of this ARISTOTLE-analagous cohort should be comparable with the results from the ARISTOTLE trial. The non-interventional analyses methods used to obtain these results may then be used to reliably estimate effects in under-studied AF patient groups.

Aims and objectives

The aims of this study are (1) to measure the association between anticoagulation treatments for stroke prevention in AF and time to stroke, systemic embolism (SE), myocardial infarction (MI), major bleeding, and mortality amongst an ARISTOTLE-analogous cohort of patients from UK electronic health records (EHR), and (2) to develop a methodological framework with in-built validation, for using observational electronic health records to answer questions about DOAC risks and benefits in patients not included in or underrepresented in the RCTs.

The specific objectives are to:

Objective 1. Check comparability of EHR data and robustness of methods for measuring stroke prevention medication effectiveness in an ARISTOTLE-analagous cohort using data from EHR data and by comparing with ARISTOTLE results.

Objective 2. Extension of trial findings: Measure treatment effects of apixaban in patient groups excluded from ARISTOTLE.

Objective 3. Comparative effectiveness: Compare treatment effectiveness between multiple individual anticoagulants (warfarin, apixaban, rivaroxaban, dabigatran) in ARISTOTLE-eligible cohorts and in patient groups excluded from ARISTOTLE.

Methods and analysis

Figure 1 (figure adapted from a study in real-world effects of medications for chronic obstructive pulmonary disease [10]) provides an overview of the study, covering the objectives and data sources used, and how RCT data will be used in Objective 1 to validate methods for analysing effectiveness of treatments for stroke prevention in AF in non-interventional data. Should Objective 1 prove successful the validated methods will be applied to unanswered questions in Objective 2 and 3.

Study design

We will use a retrospective cohort study design using longitudinal data to evaluate the effects of prescribing apixaban vs warfarin and then vs other DOACs for prevention of stroke and SE in AF on key effectiveness and safety outcomes using non-interventional primary care data.

Setting/data sources

For peer review only

Patient data used in this study will be obtained from several sources: primary care data on UK NHS patients from CPRD Gold and Aurum databases, additional data on hospital events and mortality on UK NHS patients with linked data from the Hospital Episodes Statistics (HES) and Office of National Statistics (ONS) databases, and results from the ARISTOTLE trial.

ARISTOTLE

ARISTOTLE was a randomized, double-blind trial completed in 2011, comparing apixaban with warfarin in the prevention of stroke and SE. The trial included 18,201 patients with atrial fibrillation and at least one additional risk factor for stroke. The trial was designed to test for noninferiority of apixaban compared with warfarin, and showed apixaban superiority for (1) the primary outcome of stroke or SE: hazard ratio [HR], 0.79; 95% confidence interval [CI], 0.66-0.95),[7] (2) the safety endpoint of major bleeding (HR, 0.69; 95% CI, 0.60-0.80), and (3) death from any cause (HR, 0.89; 95% CI, 0.80-0.99). The ARISTOTLE findings led to NICE guidelines on stroke prophylaxis in AF patients recommending apixaban as a treatment. Baseline patient characteristics from ARISTOTLE will be used in selection of participants in Objective 1.

CPRD Gold

CPRD Gold is a database containing anonymised data from over 625 primary care practices across the UK (approximately 13 million patient records) and is representative of the UK population with respect to age, gender and ethnicity.[11] Gold contains information on clinical diagnoses, prescribing, referrals, tests and demographic/lifestyle factors. General practices must meet prespecified standards for research-quality data to contribute data.

CPRD Aurum

CPRD Aurum contains primary care records similar to Gold but based on practices using EMIS software, whereas Gold has data from practices using Vision software. CPRD Aurum contains data on 19 million patients from 738 practices (10% of English practices) with 7 million active patients.[12]

For peer review only

Selection of participants

Participants will be selected from CPRD Gold and Aurum between 1 January 2013 and 31 July 2019. All patients will need to have been registered with a practice contributing research quality data for at least 6 months. Participant selection criteria will then vary by objective as detailed below.

Objective 1: Validation of non-interventional methods by comparing with trial results

An overview of each of the steps for participant selection for Objective 1 is provided in Figure 2.

Step 1

We will select all (HES and ONS linked) patients in the EHR cohort (CPRD Gold and Aurum) who would have met the following **inclusion** criteria for the ARISTOTLE study, at least 6 months after patient registration in the database on or prior to the index date:

- diagnosis of atrial fibrillation
- age 18+ years
- one or more stroke risk factor (age 75 years or older; prior stroke, TIA, or SE; congestive heart failure; diabetes mellitus; hypertension)

In ARISTOLE, patients randomised to apixaban were new users of apixaban whilst both treatment arms were allowed to be previous users of warfarin, with patients stratified by prior warfarin/VKA exposure. To mirror ARISTOTLE we will assess trial criteria for apixaban patients on the date of their first prescription of apixaban whilst allowing patients prescribed warfarin to become eligible at any warfarin prescription date during the study period; furthermore we will match ARISTOTLE in the proportion of new vs. prevalent users in both treatment arms. We will then exclude patients who meet any of the following ARISTOTLE study **exclusion** criteria prior to their eligible-for-inclusion date:

- AF due to reversible causes
- mitral stenosis
- increased bleeding risk
- conditions other than AF requiring chronic anticoagulation
- persistent, uncontrolled hypertension
- active infective endocarditis
- current treatment with aspirin > 165 mg/day
- simultaneous current treatment with both aspirin and a thienopyridine
- conditions likely to interfere with participation in the trial or cause death within 1 year
- recent alcohol or drug abuse, or psychosocial reasons making study participation impractical
- recent ischemic stroke (within 7 days)
- severe renal insufficiency
- ALT or AST > 2X ULN or Total Bilirubin ≥ 1.5X ULN
- platelet count ≤ 100,000/ mm3
- haemoglobin < 9 g/dL
- pregnancy or breastfeeding

For peer review only

Feasibility counts in Gold found approximately 60% of AF patients prescribed apixaban met the ARISTOTLE trial criteria. Details of the algorithms used in applying the trial criteria to the EHR data are given in the supplementary file.

Step 2

We will select a subset of apixaban patients from our EHR pool to create a cohort that matches the ARISTOTLE apixaban participants on a selection of the following baseline characteristics:

- Age
- Sex
- BMI
- Systolic blood pressure
- Congestive heart failure or left ventricular systolic dysfunction
- Hypertension requiring treatment
- Diabetes mellitus
- Prior stroke/thromboembolism
- Smoking status
- Alcohol consumption
- Level of renal impairment
- Prior VKA/warfarin exposure
- Labile INR in prior users of warfarin
- Concomitant use of: aspirin, antiplatelet or NSAID, lipid lowering drug therapy, or CYP3A4 inhibitor

This step will generate a group of ARISTOTLE-analogous apixaban patients, with similar baseline characteristics to ARISTOTLE subjects at the point of randomisation (n~9,000).

The variables selected are expected to influence the likelihood of the outcomes of interest. Exact selection of matching variables will depend on the quality and completeness of the data available and a balance will be struck between matched sample size and balance. Different methods to facilitate selection of a matched cohort will be explored, such as propensity score matching (PSM) and coarsened exact matching (CEM),[13] a nonparametric method that may give estimates with lower variance and bias for a given sample size compared than other methods[14].

Step 3

The resulting trial matched sample of EHR apixaban patients will be matched to the warfarin ARISTOTLE-eligible EHR patients (Figure 2) using a matching method such as PSM, or CEM (final method selected based upon giving optimal sample size versus balance). Risk set sampling will be employed in order to ensure similar duration of prior VKA/warfarin exposure for the prevalent users in the apixaban and warfarin EHR cohorts. The covariates for consideration in matching between EHR treatment arms or construction of a PS model will include the variables listed above in step 2 along with additional EHR variables such as data source (Gold or Aurum), socioeconomic status, and comorbidities. Each apixaban patient from the ARISTOTLE-eligible EHR patients will be matched 1:1 with the warfarin EHR patient with the closest match giving a trial-analogous cohort of ~18,000.

For peer review only - http://bmjopen.bmj.com/site/about/guidelines.xhtml

Step 4

The absolute rates and hazard ratio for the outcomes of interest (time to: stroke/SE, MI, major bleeding, and mortality) will then be calculated. For the primary outcome (time to stroke/SE) the EHR results will be validated against the ARISTOTLE trial results using the criteria detailed in Statistical Analysis (Validation of Observational Results Against Aristotle Data).

- no evidence of at least one additional risk factor for stroke

OR

- AF due to reversible causes

OR

- evidence of drug/alcohol abuse

OR

- severe comorbid condition: disease with a likelihood of causing death within 1 year or reasons making participation unpractical (such as dementia)

Objective 2: we will select patient groups who would not have been included in ARISTOTLE (and therefore would not have been included in the Objective 1 cohort) or who are under-represented in ARISTOTLE. Specifically, this will include patient groups such as patients with an AF diagnosis in the EHR cohort meeting these additional criteria:

When matching the apixaban and warfarin patients within the patient groups for this objective additional baseline variables will be considered compared with the list specified for objective 1 step 2; namely those components of the HAS-BLED score (uncontrolled hypertension, abnormal renal or liver function, and prior major bleeding or predisposition to bleeding) not included for objective 1 matching due to being ARISTOTLE exclusion criteria. In these special patient populations the same outcomes as objective 1 will be assessed, with absolute and relative rates calculated separately in each special patient group.

Objective 3: we will select all patients with AF who have a prescription for apixaban, warfarin, rivaroxaban, or dabigatran in the treatment period (between 1 January 2013 and 31 July 2019). The ARISTOTLE trial criteria will be applied followed by matching the warfarin, rivaroxaban, and dabigatran ARISTOTLE-eligible EHR patients in turn to the trial eligible EHR apixaban patients following the methodology outlined in Objective 1 Step 3. This process will result in the creation of 3 trial-eligible EHR cohorts: warfarin users matched to apixaban users, rivaroxaban users matched to apixaban users, and dabigatran users matched to apixaban users. Matched cohorts of excluded patient groups will also be constructed to enable pairwise comparisons of treatment effects in these

For peer review only

groups using the method outlined in Objective 2 above. In all cohorts the same outcomes as objective 1 will be assessed with both absolute and relative treatment effects compared.

Exposures, outcomes and co-variates

Exposures

For all objectives, exposures will be determined using CPRD Gold and Aurum prescribing records and code lists for anticoagulant treatments with no restrictions placed on the dose prescribed.

For Objectives 1 and 2, use of apixaban is the primary exposure of interest and will be compared with warfarin.

For Objective 3 other stroke prevention treatments for AF will also be compared, namely dabigatran and rivaroxaban.

Outcomes

Outcomes to be measured are as follows:

1. Stroke (ischemic or hemorraghic) or systemic embolism
2. Major bleeding
3. Myocardial Infarction
4. All cause mortality
5. Time to AF treatment change

Outcomes will be ascertained using a combination of CPRD, HES, and ONS data.

Covariates

The variables to be considered for matching patients are detailed in the selection of participants for Objective 1 (Step 2).

Sample size

Objective 1

ARISTOTLE included 9,120 patients in the apixaban arm therefore it was estimated a minimum of 15,000 EHR apixaban patients were needed for matching to be feasible. In CPRD Gold approximately 8,400 patients were eligible (January 2018). Aurum (June 2019) contained 23,526 AF apixaban patients not registered in practices that had previously contributed data to Gold. Assuming the proportion of Aurum patients meeting ARISTOTLE eligibility criteria would be similar to the proportion in Gold (~60%) gave an estimate of 14,115 trial eligible apixaban patients. Combining Gold and Aurum is therefore estimated to give >22,000 unique trial-eligible EHR apixaban patients.

For peer review only

Objectives 2 and 3

From feasibility counts we are confident we will have sufficient numbers of patients to allow well-powered analyses for objectives 2 and 3. For example, we estimate the number of people with no evidence of at least one additional risk factor for stroke for objective 2 would be >3000 people in each exposure group.

Statistical analysis

Methods of Analysis

ARISTOTLE used an intent-to-treat (ITT) approach for the primary efficacy analysis, and an on-treatment approach for sensitivity analysis and safety outcomes. We will perform equivalent analyses by using 2 different censoring schemes: a primary censoring scheme censoring 5 years after index date (reflecting the maximum possible follow-up in ARISTOTLE) for the primary effectiveness analyses, and an on-treatment scheme censoring around time of last study drug for the sensitivity analysis and safety outcome. For the on-treatment censoring scheme date of last exposure will be estimated using patient prescription data - to allow for drug half-life, stockpiling of tablets and less than 100% adherence we will add 30 days after the apparent end of treatment.

Demographic and baseline variables will be presented before and after matching steps. As the primary analysis accounts neither for treatment switching nor discontinuation, the proportion of patients discontinuing treatment and time to treatment discontinuation will be tabulated.

The primary effectiveness endpoint is time to first occurrence of confirmed stroke (ischemic, hemorrhagic, or unspecified type) or SE during the study, regardless of whether the subject is receiving treatment at the time (primary censoring scheme). Comparisons will be made according to prescribed treatment (apixaban vs warfarin).

All time to event endpoints will be analysed using a Cox proportional hazards model including treatment group as a covariate and prior warfarin/VKA status (experienced, naïve). Point estimates and two-sided 95% CIs will be constructed for the outcome. Absolute event rates of all outcomes of interest will also be calculated.

Secondary outcomes cover the key safety outcome of major bleeding and the individual outcomes of stroke, SE, MI, and mortality. Secondary outcomes other than major bleeding will use the ITT censoring scheme, major bleeding will use the on-treatment censoring scheme.

Validation of Results Against Aristotle Data

In Objective 1 alone we will validate the findings from our primary analysis against ARISTOTLE by determining whether results are compatible with the trial results. ARISTOTLE demonstrated

For peer review only

superiority of apixaban over warfarin for the primary endpoint (HR 0.79, 95% CI 0.66-0.95).[7] The treatment effect seen with EHR data may be weaker than that seen in ARISTOTLE.

An analysis of EU patients in ARISTOTLE showed a smaller treatment difference for the primary endpoint and death: HR (95% CI) for stroke/SE 0.92 (0.56-1.52), all cause death 0.89 (0.68-1.18). The European Medicines Agency Assessment Report suggested the smaller treatment effect may have been due to superior INR control in the warfarin arm of the EU subgroup (median TTR 68.93%);[15] this study could provide additional evidence on this point.

Either a result of superiority or non-inferiority will be considered compatible with ARISTOTLE results. We have set two criteria that must be met to conclude results are consistent with the trial result:

1. The effect size must be clinically comparable with the ARISTOTLE findings; the hazard ratio for time to stroke/SE with the EHR must be between 0.69 and 0.99. This range is not symmetrical around the ARISTOTLE estimate of 0.79 as it is anticipated the treatment effect in routine clinical care may be weaker than that seen in the optimised setting of a clinical trial.
2. The upper limit of the 95% CI for the rate ratio must be less than 1.52 (upper limit in the EU subgroup of ARISTOTLE).

In addition, if the upper limit of the 95% CI is less than 1 then superiority of apixaban vs warfarin will be concluded.

In order to understand the extent to which the EHR population resembles the ARISTOTLE trial population the absolute event rates of the outcomes will be compared.

Sensitivity analyses

Primary and secondary effectiveness outcomes will also be analysed using the on-treatment censoring scheme to investigate whether the extent of treatment discontinuation compromises confidence in the effectiveness analyses.

Exclusion of patient-time post treatment discontinuation in the safety and sensitivity analyses might bias results towards a conclusion of no difference[16] and risks selection bias due to attrition [17]; the set of patients who switch or discontinue treatment will therefore be examined to ascertain whether biases of this nature may have occurred.

Additional analyses may be performed using methods such as inverse-probability-of-censoring weighting (IPW) or a rank-preserving structural failure time model to estimate the treatment effect that would have been observed in the absence of treatment switching. We will explore the impact of time-varying eligibility by using methods such as a modified treatment-strategy IPW [17].

Adherence will be estimated in the EHR cohort to enable comparisons with the trial and investigate the extent to which this may have influenced differences in treatment effect observed. For apixaban

For peer review only

we will calculate the proportion of days covered (PDC) over a patient's time when on treatment as a measure of adherence. Warfarin dose is poorly recorded in EHR therefore warfarin adherence will be estimated by looking at adherence to other long-term daily medications as a proxy measure and by looking at INR control by calculating percent Time INR in Therapeutic Range (TTR) as a measure of overall warfarin treatment regime adherence.

We will perform a supplementary analysis in patients deemed adherent (PDC ≥ 80% matching ARISTOTLE compliance limit) along with an exploratory subgroup analysis by INR TTR. The different nature of the proxy variables used for adherence in the DOACs (PDC) compared with warfarin (INR TTR) means that the adherence estimates may not be comparable; should great differences in adherence be observed between these exposure groups the definitions of adherence used may need to be reassessed.

Apixaban was a newly available drug with a low number of patients having a prescription in the first year it was available [18]; we will therefore perform a sensitivity analysis with the start of the study period shifted forwards a year to January 2014 to investigate the impact of inclusion of early adopters who may differ from later adopters of a new drug.

Plan for addressing confounding

In the study period apixaban was a newly available treatment leading to the possibility of channelling bias. For objective 1 by applying trial eligibility criteria to both treatment cohorts and matching using the baseline covariates we should avoid channelling bias. To handle confounding treatment arms will be matched using the optimal method selected. Unmeasured or unknown confounding may remain and this will be explored in the analysis and discussion of results.

Missing Baseline Data

UK EHR data have been shown to be almost complete for drug prescribing and information on important comorbidity are well recorded. For some variables such as renal function and alcohol intake, a patient is more likely to have no data entered if there is no overt clinical evidence of abnormality; in such cases we may take a pragmatic approach categorising into a parameter ("evidence of" vs "no evidence of") with those with no data included in the "no evidence of" group. For BMI and SBP we cannot assume data are missing at random as we expect a patient is less likely to have these recorded if they appear healthy weight and do not have hypertension respectively or if they have a lower comorbidity burden. Furthermore, as the proportion of patients with missing baseline BMI or SBP is expected to be low (approximately 4% for BMI and <1% for SBP [18]) these patients will be excluded from the trial-eligible cohort.

Missing Prescription Data

Treatment may be initiated in secondary care meaning the first prescription of patients newly

For peer review only

initiating treatment or switching treatments are missing; to account for this we will perform a sensitivity analysis where those newly initiating treatment are assigned an earlier derived index date. Hospitalised patients may have prescriptions in secondary care leading to treatment gaps in their primary care data. We will investigate the occurrence of hospitalisation around treatment discontinuation and assess the potential impact on the results of missed events by performing a sensitivity analysis with different extended derived dates of last dose. Some concomitant drugs used in determining eligibility and matching patients are available over the counter meaning we may miss that patients are exposed to these; we expect OTC use of these drugs to be similar in both treatment groups.

Missing Outcome Data

EHR data are shown to be almost complete for mortality.[19] Patient deaths missing from EHRs are expected to be missing at random equally in both treatment arms thereby not altering the overall direction of treatment effect. The classification of unspecified stroke type will cause uncertainty in the main safety endpoint and may lead to a lower event rate for major bleeding compared with the trial; this would affect the power but should not affect the treatment effect seen as events are expected to be missing at random from both treatment arms.

Limitations of the study design, data sources, and analytic methods

Some of the criteria assessed for ARISTOTLE eligibility may not be well recorded in CPRD, criteria such as alcohol and drug abuse may not be captured for all patients. For criteria such as "increased bleeding risk" it is unclear which codes to include and time scale to consider. These limitations are consistent with our aim to select a population as similar as possible to ARISTOTLE whilst acknowledging differences will remain. The most important risk factors for the primary outcome of stroke (the components of CHA2DS2-VASc score for AF stroke risk) are mostly well recorded in CPRD.[20]

There are differences in the coding systems used by the two EHR data sources and completeness of coding may differ between the two; the potential impact of this will be ascertained by comparisons of rates of diagnoses, baseline variables, and prescriptions of interest. Inclusion of data source as a matching variable should prevent discrepancy between the sources from biasing results. We will explore different methods of combining Gold and Aurum, namely analysing separately by database and combining the results as a metanalysis as an alternative to combining data before analysis.

The main focus of the study is validation of our methodology through assembling a cohort of patients comparable to the patients in ARISTOTLE and finding similar results to the trial. Criteria to determine the success of the methodology have been pre-specified in the protocol. Given the use of CPRD data to determine treatment effectiveness is not yet well established, a finding that these data are not suitable to answer questions on intended effectiveness will be a useful conclusion.

For peer review only

Patient and Public Involvement

No patient involved.

Ethics and dissemination

Approval by ethics and scientific comittees

An application for scientific approval related to use of CPRD data was approved by the Independent Scientific Advisory Committee (ISAC) of the Medicines and Healthcare Products Regulatory Agency (MHRA).

Dissemination plans

The results of the study will be submitted to peer reviewed journals and presented at conferences. Relevant charities will be contacted for guidance on dissemination of results to patients in an accessible manner. We will communicate with NICE to convey any results relevant to the guidance they have issued on AF, and with the MHRA if findings may impact the risk/benefit profile of anticoagulation treatments in AF patients.

For peer review only

Contributor statement

EP, KW, ID, UG, LS contributed to study question and design. EP wrote the first draft of the protocol manuscript (based upon original proposal to MRC, ISAC that EP, KW, ID, UG and LS all contributed to). EP, KW, ID, UG, LS contributed to further drafts and approved the final version.

Competing interests

Ms Powell and Dr Wing declare no competing interests. Professor Douglas reports grants from GlaxoSmithKline, NIHR, ABPI, MRC and holds stock in GlaxoSmithKline. Professor Smeeth reports grants from Wellcome, MRC, NIHR, BHF, Diabetes UK, ESRC and the EU; grants and personal fees for advisory work from GSK, and historical personal fees for advisory work from AstraZeneca. He is a Trustee of the British Heart Foundation. Ms Gungabissoon is an employee of and holds shares in GlaxoSmithKline.

Funding

This work was supported by the Medical Research Council through a MRC LID studentship [grant number MR/N013638/1]

Data sharing statment

There are currently no unpublished data from this study as it is a protocol. All of the data sources described can be accessed by making formal applications to the owners of the data (CPRD/HES data

for the routinely collected non-interventional data and Bristol-Myers Squibb for the trial summary and results used for validation of non-interventional methods).

References

1 Adderley NJ, Ryan R, Nirantharakumar K, *et al.* Prevalence and treatment of atrial fibrillation in UK general practice from 2000 to 2016. *Heart* 2019;**105**:27–33. doi:10.1136/HEARTJNL-2018-312977

2 Davis RC, Hobbs FDR, Kenkre JE, *et al.* Prevalence of atrial fibrillation in the general population and in high-risk groups: the ECHOES study. *Europace* 2012;**14**:1553–9. doi:10.1093/europace/eus087

3 Wolf PA, Abbott RD, Kannel WB. Atrial fibrillation as an independent risk factor for stroke: The framingham study. *Stroke* 1991;**22**:983–8. doi:10.1161/01.STR.22.8.983

4 Brønnum-Hansen H, Davidsen M, Thorvaldsen P. Long-term survival and causes of death after stroke. *Stroke* 2001;**32**:2131–6. doi:10.1161/hs0901.094253

5 Adamson J, Beswick A, Ebrahim S. Is stroke the most common cause of disability? *J Stroke Cerebrovasc Dis* 2004;**13**:171–7. doi:10.1016/j.jstrokecerebrovasdis.2004.06.003

6 Nelson WW, Wang L, Baser O, *et al.* Out-of-range INR values and outcomes among new warfarin patients with non-valvular atrial fibrillation. *Int J Clin Pharm* 2014;**37**:53–9. doi:10.1007/s11096-014-0038-3

7 Granger CB, Alexander JH, McMurray JJV, *et al.* Apixaban versus Warfarin in Patients with Atrial Fibrillation. *N Engl J Med* 2011;**365**:981–92. doi:10.1056/NEJMoa1107039

8 U.S. Department of Health and Human Services F and D. Guidance for Industry Postmarketing Studies and Clinical Trials — Implementation of Section 505(o)(3) of the Federal Food, Drug, and Cosmetic Act.

9 EUROPEAN PARLIAMENT AND THE COUNCIL OF THE EUROPEAN UNION. REGULATION (EU) No 1235/2010 OF THE EUROPEAN PARLIAMENT AND OF THE COUNCIL of 15 December 2010.

10 Wing K, Williamson E, Carpenter JR, *et al.* Real-world effects of medications for chronic obstructive pulmonary disease: protocol for a UK population-based non-interventional cohort study with validation against randomised trial results. *BMJ Open* 2018;**8**:e019475. doi:10.1136/bmjopen-2017-019475

11 Herrett E, Gallagher AM, Bhaskaran K, *et al.* Data Resource Profile: Clinical Practice Research Datalink (CPRD). *Int J Epidemiol* 2015;**44**:827–36. doi:10.1093/ije/dyv098

For peer review only

12 Wolf A, Dedman D, Campbell J, *et al.* Data resource profile: Clinical Practice Research Datalink (CPRD) Aurum. *Int J Epidemiol* Published Online First: 11 March 2019. doi:10.1093/ije/dyz034

13 Iacus SM, King G, Porro G. Causal Inference without Balance Checking: Coarsened Exact Matching. *Polit Anal* 2012;**20**:1–24. doi:10.1093/pan/mpr013

14 King G, Nielsen R, Coberley C, *et al.* Comparative Effectiveness of Matching Methods for Causal Inference. https://gking.harvard.edu/publications/comparative-effectiveness-matching-methods-causal-inference (accessed 4 Sep 2018).

15 European Medicines Agency. Committee for Medicinal Products for Human Use (CHMP). Assessment report Eliquis apixaban. Procedure No.:EMEA/H/C/002148/X/04/G. EMA/641505/2012. 2012.

16 Jones B, Jarvis P, Lewis JA, *et al.* Trials to assess equivalence: the importance of rigorous methods. *BMJ* 1996;**313**:36–9.http://www.ncbi.nlm.nih.gov/pubmed/8664772 (accessed 4 Sep 2018).

17 Schnitzer ME, Platt RW, Durand M. A tutorial on dealing with time-varying eligibility for treatment: Comparing the risk of major bleeding with direct-acting oral anticoagulant <scp>s</scp> vs warfarin. *Stat Med* 2020;**39**:4538–50. doi:10.1002/sim.8715

18 Vinogradova Y, Coupland C, Hill T, *et al.* Risks and benefits of direct oral anticoagulants versus warfarin in a real world setting: cohort study in primary care. *BMJ* 2018;**362**:k2505.

19 Gallagher AM, Williams T, Leufkens HGM, *et al.* The Impact of the Choice of Data Source in Record Linkage Studies Estimating Mortality in Venous Thromboembolism. *PLoS One* 2016;**11**:e0148349. doi:10.1371/journal.pone.0148349

20 Khan NF, Harrison SE, Rose PW. Validity of diagnostic coding within the General Practice Research Database: a systematic review. *Br J Gen Pract* 2010;**60**:e128–36. doi:10.3399/bjgp10X483562

Figure 1: Overview of study objectives and sources of data for the real-world effects of medications for stroke prevention in AF study

(RCT=randomised controlled trial, CPRD= the UK Clinical Practice Research Datalink, AF=Atrial Fibrillation, EHR= Electronic Healthcare Records)

For peer review only

Figure 2: Flowchart illustrating the Assembly of a Matched Trial-analogous Cohort of EHR Patients

EHR= Electronic Health Record; CPRD= Clinical Practice Research Datalink; AF= Atiral fibrillation

For peer review only

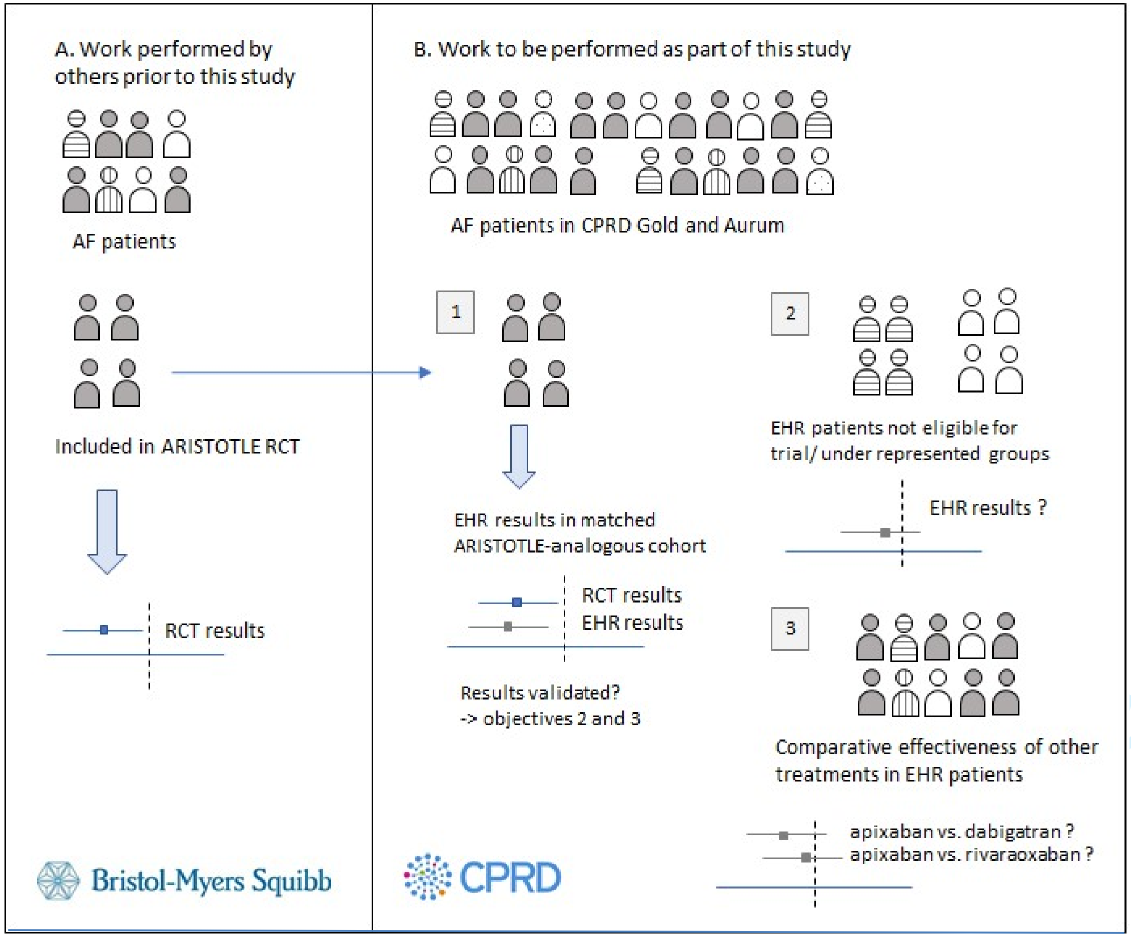

A. Work performed by others prior to this study

ARISTOTLE: RCT that investigated effectiveness and safety of apixaban vs warfarin in prevention of stroke and systemic embolism in AF patients. RCTs results inform clinical practice despite only a subset (based on trial inclusion and exclusion criteria) of the total population of AF patients being included in the RCTs of stroke prophylaxis treatments.

B. Work to be performed as part of this study

<u>1. Objective 1</u>

A cohort of ARISTOTLE-analogous patients will be selected from UK EHRs (CPRD Gold and Aurum), by matching EHR patients prescribed apixaban to the apixaban patients included in the trial on baseline characteristics. EHR patients prescribed warfarin will then be matched to the trial-analagous EHR apixaban patients. An analysis of the effectiveness of apixaban vs. warfarin on prevention of stroke/systemic embolism will then be performed on this ARISTOTLE-analagous EHR cohort. If the results obtained are comparable to those obtained in ARISTOTLE, this will serve as a validation step, showing that data from the non-inverventional CPRD Gold and Aurum sources can reliably be used to study stroke prevention treatment effects in AF.

<u>2. Objective 2</u>

The validated analysis techniques used for Objective 1 will then be used to study UK EHR patients who would not have been eligible for inclusion in an RCT or are under-represented in RCTs due to their age or presence of other comorbidities, for whom the comparative effects of anticoagulants in stroke prevention in AF is unclear.

<u>3. Objective 3</u>

The validated analysis techniques used for Objective 1 will then be used to compare effectiveness of apixaban vs warfarin, apixaban vs rivaroxaban and apixaban vs dabigatran.

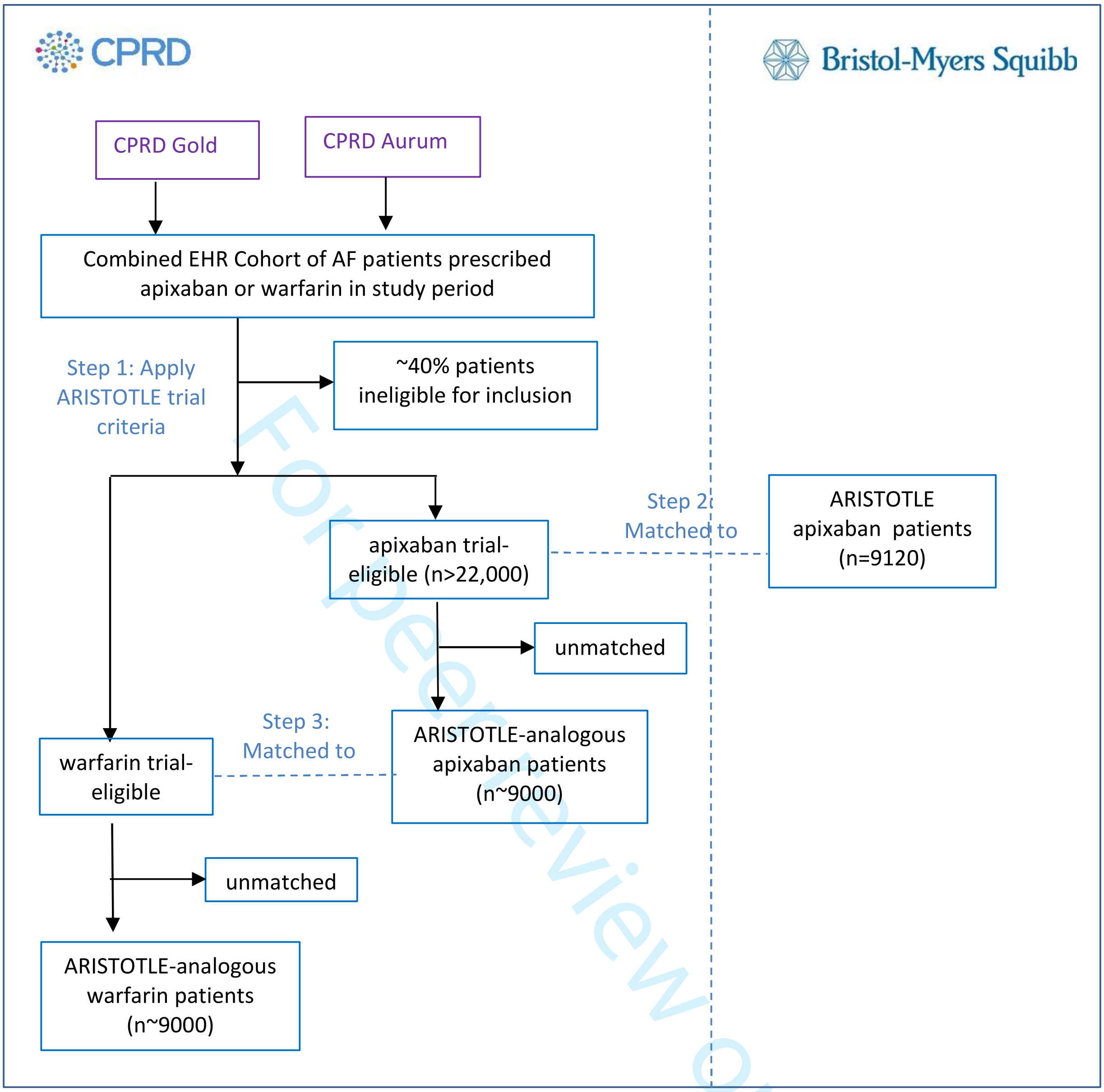

For peer review only

Appendix Table: ARISTOTLE Inclusion and Exclusion Criteria Algorithms for EHR

To be trial eligible a patient must have all inclusion criteria (IE01 to IE03)=Y and no exclusion (IE05 to IE27c)=Y

Criteria #	Used?	Criteria Text (from ARISTOTLE protocol)	Implementation Rule and Notes
		Inclusion Critera (IE01 to IE04a)	
IE01	Y	Age ≥ 18 years	Calculate age at index date, day and month of birth not available therefore calculate age by assuming birthdate=01-July-birthyear:<br>age =(indexdate-birthdate)/365.25<br><br>If age ge 18 then IE01=Y.
IE02	Y	In atrial fibrillation or atrial flutter not due to a reversible cause and documented by ECG at the time of enrollment. OR If not in atrial fibrillation/flutter at the time of enrollment, must have atrial fibrillation/flutter documented on two separate occasions, not due to a reversible cause at least 2 weeks apart in the 12 months prior to enrollment. Atrial fibrillation/flutter may be documented by ECG, or as an episode lasting at least one minute on a rhythm strip, Holter recording, or intracardiac electrogram (from an implanted pacemaker or defibrillator).	If patient has medical record corresponding to atrial fibrillation or atrial flutter on or prior to index date then IE02=Y.
		One or more of the following risk factor(s) for stroke:	IE03=Y if at least one of (IE03a, IE03b, IE03c, IE03d, IE03e) is Y.
IE03a	Y	Age 75 years or older	See IE01 for derivation of age at index date.<br>If age ge 75 then IE03a=Y
IE03b	Y	Prior stroke, TIA or systemic embolus	If patient has medical record corresponding to stroke, TIA, or systemic embolus diagnosis on or prior to index date then IE03b=Y.<br><br>Codelist search terms include 'stroke', 'cerebrovascular accident', 'cerebral infarction', 'lacunar', 'transient ischaemic attack', and synonyms for these.
IE03c	Y	Either symptomatic congestive heart failure within 3 months or left ventricular dysfunction with an LV ejection fraction (LVEF) ≤ 40% by echocardiography, radionuclide study or contrast angiography	If patient has medical record corresponding to congestive heart failure or left ventricular dysfunction diagnosis on or prior to index date then IE03c=Y.<br><br>Codelist search terms include 'heart failure', 'cardiac failure', 'congestive heart failure', 'cardiomyopathy', 'left ventricular dysfunction', 'left ventricular', 'lvef', 'new york heart association classification', 'hypertensive heart', and synonyms for these.
IE03d	Y	Diabetes mellitus	If patient has medical record corresponding to diabetes diagnosis on or prior to index date then IE03d=Y.<br><br>Codelist search terms include ‘diabetes’, both type 1 and type 2 diabetes are included.

Criteria #	Used?	Criteria Text (from ARISTOTLE protocol)	Implementation Rule and Notes
IE03e	Y	Hypertension requiring pharmacological treatment	If patient has medical record corresponding to hypertension on or prior to index date AND a prescription for an antihypertensive on or prior to index date then IE03e=Y.<br><br>Hypertension codelist search terms include 'hyperten', 'high blood pressure', 'nephrosclerosis', and synonyms for these.
IE04	N	Women of childbearing potential (WOCBP) must be using an adequate method of contraception to avoid pregnancy throughout the treatment period of the study or for 2 weeks after the last dose of study medication, whichever is longer, in such a manner that the risk of pregnancy is minimized. WOCBP must have a negative serum or urine pregnancy test (minimum sensitivity 25 IU/L or equivalent units of HCG) within 48 hours prior to the start of investigational product.	This criteria is only partially applied - women with evidence of recent pregnancy or breastfeeding will be excluded (see IE27c).
IE04b	N	All subjects must provide signed written informed consent.	N/A for observational study
		Exclusion criteria (IE05 to IE27d)	
IE05	Y	Atrial fibrillation or flutter due to reversible causes (e.g. thyrotoxicosis, pericarditis)	If patient has medical record corresponding to reversible AF causes on or prior to index date then IE05=Y.<br><br>Codelist search terms include 'thyrotoxicosis', 'pericarditis', and synonyms for these.
IE06	Y	Clinically significant (moderate or severe) mitral stenosis	If patient has medical record corresponding to mitral stenosis on or prior to index date then IE06=Y.<br><br>Cannot determine clinical significance of 'mitral stenosis' terms in CPRD therefore assume if there is a record of mitral stenosis the condition is clinically significant.
IE07	Y	Increased bleeding risk that is believed to be a contraindication to oral anticoagulation (e.g. previous intracranial hemorrhage)	If patient has medical record corresponding to increased bleeding risk on or prior to index date then IE07=Y.<br><br>Codelist search terms include 'haemorrhag', 'bleed', 'aneurysm', (('intracranial' or 'brain') and ('neoplasm' or 'tumour' or 'cancer')), 'arteriovenous malformation', 'immune thrombocytopenic purpura', 'evans disease', 'hemolytic anemia', 'haemophilia', 'von willebrand disease', ('glanzmann' and 'thrombasthenia'), 'wiskott–aldrich syndrome', 'thrombocytopenia' and synonyms for these.<br><br>For some forms of more common past bleeding event such as bleeding related to menstrual or uterine bleeding, bleeding associated with surgery or injury, bleeding associated with ulcer or gastritis, eye bleeding (retinal, conjunctival) we apply the additional criteria that these must be within the last two years to be included as evidence of increased bleeding risk.

For peer review only

Criteria #	Used?	Criteria Text (from ARISTOTLE protocol)	Implementation Rule and Notes
IE08	Y	Conditions other than atrial fibrillation that require chronic anticoagulation (e.g. prosthetic mechanical heart valve)	If patient has medical record corresponding to a condition other than atrial fibrillation that requires chronic anticoagulation on or prior to index date then IE08=Y.<br><br>Codelist search terms include (('heart' or 'valve') and ('prosth' or 'mechanical')), 'venous thromb', and synonyms for these.
IE09	Y	Persistent, uncontrolled hypertension (systolic BP > 180 mm Hg, or diastolic BP > 100 mm Hg)	If patient has at least 2 blood pressure readings over the limit (systolic BP > 180 mm Hg, or diastolic BP > 100 mm Hg) in the 6 months prior to the index date<br>OR<br>the patient has a medical record (within 180 days prior to index date) indicating uncontrolled hypertension then IE09=Y<br><br>Codelist search terms include 'poor hypertension control', 'hypertensive crisis', 'malignant hypertension', 'severe hypertension', 'hypertension resistant to drug therapy', and synonyms for these.
IE10	Y	Active infective endocarditis	If patient has medical record corresponding to endocarditis on or prior to index date then IE10=Y.
IE11	N	Planned major surgery	N/A – do not look at future events when determining eligibility
IE12	N	Planned atrial fibrillation or flutter ablation procedure	N/A – do not look at future events when determining eligibility
IE13	N	Use of an unapproved, investigational drug or device within the past 30 days	N/A – not appropriate to apply when looking at observational data
IE14	Y	Required treatment with aspirin > 165 mg/day	If patient has a prescription for aspirin with dose > 165 mg/day and prescription data suggests drug exposure ongoing at index date then IE14=Y.<br>Note this will not pick up patients taking regular aspirin over the counter (study limitation).
IE15	Y	Simultaneous treatment with both aspirin and a thienopyridine (e.g., clopidogrel, ticlopidine)	If both aspirin and thienopyridine ongoing at index date (ie derived exposure covers index date) then IE15=Y.
IE16	Y	Severe comorbid condition with life expectancy of ≤ 1 year	If patient has medical record corresponding to a condition with a low median survival time then IE16=Y.<br>Codelist search terms include pancreatic, oesophageal, stomach, liver, gallbladder, biliary duct, bladder, lung or brain cancer, multiple myeloma, mesothelioma, CJD, and synonyms for these.
IE17	Y	Active alcohol or drug abuse, or psychosocial reasons that make study participation impractical	If patient has medical record corresponding to drug or alcohol abuse or any complications of abuse, conditions involving an impaired mental state (dementia including subtypes such as Alzheimer's), severe mental health conditions (schizophrenia, psychosis, bipolar) then IE17=Y.
IE18	Y	Recent ischemic stroke (within 7 days)	If patient has medical record corresponding to ischemic stroke within 7 days of index date (prior) then IE18=Y.

For peer review only

Criteria #	Used?	Criteria Text (from ARISTOTLE protocol)	Implementation Rule and Notes
IE19	Y	Severe renal insufficiency (serum creatinine > 2.5 mg/dL or a calculated creatinine clearance < 25 mL/min, See Section 6.3.2.2)	If patient has lab result showing serum creatinine > 2.5 mg/dL or a calculated creatinine clearance < 25 mL/min within 90 days prior to index date<br>OR<br>a medical record corresponding to severe renal insufficiency (chronic kidney disease stage 4 or 5, dialysis)<br>then IE19=Y
IE20	Y	ALT or AST > 2X ULN or a Total Bilirubin ≥ 1.5X ULN (unless an alternative causative factor [e.g., Gilbert's syndrome] is identified)	If patient has lab result showing ALT or AST > 2X ULN or a Total Bilirubin ≥ 1.5X ULN within 90 days prior to index date (AND no diagnosis of Gilbert's syndrome) then IE20=Y
IE21	Y	Platelet count ≤ 100,000/ mm3	If patient has lab result showing platelet count ≤ 100,000/ mm3 within 90 days prior to index date<br>OR<br>a medical record of thrombocytopenia within 90 days prior to index date<br>then IE21=Y
IE22	Y	Hemoglobin < 9 g/dL	If patient has lab result showing hemoglobin < 9 g/dL within 90 days prior to index date then IE22=Y
IE23	N	Inability to comply with INR monitoring	Patients unlikely to be able to comply with INR monitoring – evidence of drug or alcohol abuse, impaired mental state, severe mental health conditions. All these conditions are already excluded by IE17
IE24	N	Prior randomization into an apixaban clinical study	N/A
IE25	N	Prisoners or subjects who are involuntarily incarcerated	N/A
IE26	N	Subjects who are compulsorily detained for treatment of either a psychiatric or physical (e.g., infectious disease) illness	N/A
	N	Women of child bearing potential (WOCBP) unwilling or unable to use an acceptable method to avoid pregnancy:	N/A – see IE27c
IE27a	N	WOCBP using a prohibited contraceptive method	N/A

For peer review only - http://bmjopen.bmj.com/site/about/guidelines.xhtml

Criteria #	Used?	Criteria Text (from ARISTOTLE protocol)	Implementation Rule and Notes
IE27b	N	WOCBP include any female who has experienced menarche and who has not undergone successful surgical sterilization (hysterectomy, bilateral tubal ligation, or bilateral oophorectomy) or is not postmenopausal [defined as amenorrhea ≥ 12 consecutive months, or women on hormone replacement therapy (HRT) with documented serum follicle stimulating hormone (FSH) level > 35 mIU/mL]. Even women who are using oral contraceptives, other hormonal contraceptives (vaginal products, skin patches, or implanted or injectable products), or mechanical products such as an intrauterine device or barrier methods (diaphragm, condoms, spermicides) to prevent pregnancy, or are practicing abstinence or where their partner is sterile (e.g., vasectomy) should be considered to be of child bearing potential	N/A
IE27c	Y	Women who are pregnant or breastfeeding	Exclude women who have any medical codes relating to pregnancy (regardless of the outcome of the pregnancy), childbirth, antenatal or postnatal care, or breastfeeding in the 3 years prior to the patient's index date.
IE27d	N	Women with a positive pregnancy test on enrollment or prior to administration of investigational product.	N/A – covered by IE27c

Note: Algorithms are under development as part of this study and may be further refined prior to being finalised.
N/A = Not Applicable. For IE19-IE22 involving lab results a pragmatic approach will be taken in which a patient is assumed not to have the exclusion criteria if there is no lab result available in the 90 days prior to index date and the latest available lab result prior to index date does not meet the criteria.

For peer review only
